# Genome-wide characterization of *GRAS* family genes in *Medicago truncatula* reveals their evolutionary dynamics and functional diversification

**DOI:** 10.1371/journal.pone.0185439

**Published:** 2017-09-25

**Authors:** Hailing Zhang, Yingping Cao, Chen Shang, Jikai Li, Jianli Wang, Zhenying Wu, Lichao Ma, Tianxiong Qi, Chunxiang Fu, Zetao Bai, Baozhong Hu

**Affiliations:** 1 College of Life Science, Northeast Agricultural University, Harbin, China; 2 Pratacultural Sciences Institute of Heilongjiang Academy of Agricultural Sciences, Harbin, Heilongjiang, China; 3 Key Laboratory of Biofuels, Shandong Provincial Key Laboratory of Energy Genetics, Qingdao Institute of Bioenergy and Bioprocess Technology, Chinese Academy of Sciences, Qingdao, China; 4 Qingdao Engineering Research Center of Biomass Resources and Environment, Qingdao Institute of Bioenergy and Bioprocess Technology, Chinese Academy of Sciences, Qingdao, China; 5 Harbin University, Harbin, China; Clemson University, UNITED STATES

## Abstract

The *GRAS* gene family is a large plant-specific family of transcription factors that are involved in diverse processes during plant development. *Medicago truncatula* is an ideal model plant for genetic research in legumes, and specifically for studying nodulation, which is crucial for nitrogen fixation. In this study, 59 *MtGRAS* genes were identified and classified into eight distinct subgroups based on phylogenetic relationships. Motifs located in the C-termini were conserved across the subgroups, while motifs in the N-termini were subfamily specific. Gene duplication was the main evolutionary force for *MtGRAS* expansion, especially proliferation of the LISCL subgroup. Seventeen duplicated genes showed strong effects of purifying selection and diverse expression patterns, highlighting their functional importance and diversification after duplication. Thirty *MtGRAS* genes, including *NSP1* and *NSP2*, were preferentially expressed in nodules, indicating possible roles in the process of nodulation. A transcriptome study, combined with gene expression analysis under different stress conditions, suggested potential functions of *MtGRAS genes* in various biological pathways and stress responses. Taken together, these comprehensive analyses provide basic information for understanding the potential functions of *GRAS* genes, and will facilitate further discovery of *MtGRAS* gene functions.

## Introduction

Transcription factors play important roles in regulating various plant development and physiological processes. The plant-specific *GRAS* gene family has been studied in nearly 30 plant species from more than 20 genera [1, 2]. Evolutionary analyses have suggested that the *GRAS* gene family possibly originated from bacteria through horizontal gene transfer [3]. The name *GRAS* is derived from the first three transcription factors identified in this family: gibberellic-acid insensitive (*GAI*) [4], repressor of GAI (*RGA*) [5], and scarecrow (*SCR*) [6]. GRAS proteins typically contain 400–770 amino acids [7]. Their C-terminal regions are highly conserved, and include several ordered motifs, namely leucine heptad repeat I (LHRI, LRI), VHIID, leucine heptad repeat II (LHRII, LRII), PFYRE, and SAW, which are crucial in interactions between GRAS and other proteins [8]. In *Arabidopsis*, mutation of the SAW and PFYRE motifs in SCR1 and RGA proteins resulted in distinct phenotypic variation [5]. However, the length and sequence of N-terminal regions in GRAS proteins are more divergent, indicating their potential function in protein specificity [1].

Many studies have defined categories of GRAS proteins [1, 9–13]. According to a study in *Arabidopsis thaliana* and rice (*Oryza sativa* L.), the *GRAS* family is divided into 8 sub-branches, including LISCL, PAT1, SCL3, DELLA, SCR, SHR, LAS, and HAM [14]. However, in other studies in poplar (*Populus trichocarpa* L.), bean (*Ricinus communis* L.), and tomato (*Solanum lycopersicum* L.), the number of distinct clades ranged from 8 to 13 [12, 15, 16]. A large number of *GRAS* genes have been functionally characterized in various species [17–21]. In lily (*Lilium longiflorum* L.), *LiSCL* (LISCL subfamily) participated in microsporogenesis of anthers [22]. In *Arabidopsis*, SCL14 (LISCL subfamily) interacted with TGA transcription factors to activate a broad-spectrum detoxification network [23]. Three *Arabidopsis* genes, *PAT1*, *SCL5*, and *SCL21* (PAT1 subfamily), are positive regulators in phytochrome-A signal transduction [24, 25], whereas *SCL13* (PAT1 subfamily) is mainly involved in phytochrome-B signal transduction [26]. GAI, RGA, and RGL (DELLA subfamily) proteins mainly function as repressors of gibberellin signaling [19]. SCR and SHR form a SCR/SHR complex, which plays an essential role in root radial patterning [27]. SCL3 acts downstream of the GA/DELLA and SCR/SHR pathways, and mediates cell elongation during root development [28]. The role of *MOC1*, *LS*, and *LAS* (LAS subfamily) in axillary meristem initiation has been validated [29–31]. The *microRNA171*(*miR171*) family is one of the most ancient and well conserved miRNA families which have diverse roles in plant development, such as flowering, meristem identity, and phase transition [32, 33]. Overexpressing *miR171* had pleiotropic phenotypes including plant height, flowering time, leaf architecture, phase transitions and floral meristem determinacy [34–36]. This family is known to target the HAM genes. Three *HAM* homologs in *Arabidopsis* (*SCL6-II*, *SCL6-III*, and *SCL6-IV*) were post-transcriptionally regulated by *miRNA171* and play vital roles in the proliferation of meristematic cells [37–39]. Furthermore, the triple *scl6* mutants and overexpressing *miR171* showed similar pleiotropic phenotypes [34].

*Medicago truncatula* is an annual, diploid legume plant. Because of its small genome size, self-pollination, and a well-established transformation platform, *M*. *truncatula* is an ideal model for genetic studies of legumes. The genome sequence of *M*. *truncatula* was released in 2011 and was recently updated, which provides the opportunity for gene family analysis on a genomic level [40]. To absorb nitrogen, leguminous plants have established a symbiotic relationship with nitrogen fixing rhizobial bacteria, forming special lateral organs called nodules. Certain types of transcription factors have been reported to play crucial roles in regulating the nodulation including GRAS, AP2/ERF, and NF-Y [41, 42]. *Medicago truncatula* can serve as a model for the molecular mechanism mediating nodulation, which is very important for understanding nitrogen acquisition and fixation in legumes [43]. Recent studies have suggested that GRAS proteins are involved in initiating nodulation [2]. *NSP1* and *NSP2*, two GRAS proteins in *M*. *truncatula*, play an essential role in nodule morphogenesis, serving as possible regulators of Nod-factor-inducible gene expression [44]. To explore the functional role of *GRAS* genes, especially in the process of nodulation, we performed a genome-wide study of the entire *GRAS* gene family in *M*. *truncatula*.

Genomic analyses of the *GRAS* gene family have been conducted in various species including *Arabidopsis* [1], rice [1], Chinese cabbage (*Brassica rapa* ssp. *pekinensis*) [9], tomato [12], castor beans [16] and grapevine (*Vitis vinifera* L.) [10], but have not been explored in legumes. In this study, we systematically and comprehensively analyzed *GRAS* genes in *M*. *truncatula* using comparative genomic strategies and experimental validation. The aims of this study were as follows: (1) identify and classify *GRAS* genes in *M*. *truncatula*; (2) explore the evolutionary dynamics of *MtGRAS* gene proliferation and uneven distribution; and (3) determine the functional diversity of *MtGRAS* genes by structure conservation analysis and expression profile analysis in different tissues and stress treatments. These findings provide insights into the molecular functions of *MtGRAS* genes, and will be helpful for future functional characterization of *GRAS* genes in legumes.

## Materials and methods

### Identification of *MtGRAS* genes

The current genome sequence and annotation files (Mt4.0v1) of *M*. *truncatula* were downloaded from Phytozome (https://phytozome.jgi.doe.gov/pz/portal.html). The most updated Hidden Markov Model (HMM) for the *GRAS* gene family, PF03514.11, was downloaded from the Pfam database (http://pfam.xfam.org). Using PF03514.11 as a query, we conducted a BLAST search against the entire protein dataset of *M*. *truncatula* with a cut-off E-value of 1e-10 using the blastall v2.2.18 package. Subsequently, all hit protein sequences were extracted using custom Perl scripts. Then, the integrity of the GRAS domain was evaluated using SMART tools [45], and candidate MtGRAS proteins composed of a truncated GRAS domain were identified. To obtain the gene structure, a GFF3 annotation file involving precise position information of introns and exons in each *MtGRAS* was retrieved from the genomic dataset, and uploaded to the Gene Structure Display Server (http://gsds.cbi.pku.edu.cn/) [46]. Peptide length, molecular weight, and isoelectric point of each MtGRAS protein were calculated using the online ExPasy program (http://www.expasy.org/) [47].

### Classification and conservation analysis of *MtGRAS* genes

The identified MtGRAS proteins were combined with the well-classified *Arabidopsis* and rice GRAS proteins and aligned using ClustalW [48]. Then, a phylogenetic tree was constructed in MEGA5 software using the neighbor-joining method with 1000 bootstrap replicates [49]. The phylogenetic tree was visualized using Evolview (http://www.evolgenius.info/evolview/) [50]. The *MtGRAS* genes were further categorized into different subgroups according to homology with *GRAS* genes in *Arabidopsis* and rice. The conserved motif analysis of *MtGRAS* was conducted using the motif finding tool, MEME (Multiple EM for Motif Elicitation, v4.10.0) with 20 motif numbers, and the order of motifs in each *MtGRAS* was evaluated by MAST [51]. The targets of *miR171* were predicted *in silico* using the website (http://plantgrn.noble.org/psRNATarget/). The *Pv-miR171* genes were identified based on the homology searching stem-loop sequence of *osa-miR171*, which obtained from the website of miRbase (http://www.mirbase.org/index.shtml).

### Chromosomal distribution and gene duplication analysis of *MtGRAS* genes

Physical positions of *MtGRAS* genes were retrieved from the GFF3 annotation file using a Perl script, and diagrams of their chromosomal locations and duplication events were drawn using Circos software (http://circos.ca/) [52]. Homologous gene pairs were defined as having protein similarity of more than 70% and coverage greater than 75%. In addition, gene duplication information was also identified based on public data in the Plant Genome Duplication Database (PGDD, http://chibba.agtec.uga.edu/duplication/) [53]. If two homologous genes were separated by five or fewer genes, they were identified as tandem duplications (TD), while if two genes were separated by more than five genes or distributed in different chromosomes, they were referred to as segmental duplications (SD). To determine the evolutionary pressure acting on duplicated genes, Ka (non-synonymous substitution) and Ks (synonymous substitution) values were calculated using KaKs_Calculator 2.0 [54].

### Expression profile analysis of *MtGRAS* genes using RNA-seq

Illumina single read sequencing data for the transcriptome of *M*. *truncatula* were obtained from the NCBI Short Read Archive (accession numbers SRX099059-SRX099062). This dataset contained six different tissues including root, flower, bud, seedpod, blade, and nodule. We aligned all reads from each tissue to the reference genome of *M*. *truncatula* (Mt4.0v1) using tophat v2.1.0 [55]. Subsequently, the expression level for each *MtGRAS* was measured using Cufflink v2.1.1 [56], and the FPKM (fragment per kilobase per million mapped reads) representing the gene expression level of each *MtGRAS* was extracted with custom Perl scripts. A heatmap of the *MtGRAS* expression profile was created using Mev v4.8.1 [57].

### Biotic and abiotic stress treatments

For hormone treatment, three-week-old *M*. *truncatula* (cv. Jemalong A17) seedlings were soaked in liquid MS medium with 30 μM gibberellin (GA3). For cold treatment, seedlings were grown in a greenhouse (12/12h photoperiod, 18–24°C) and transferred to a cold chamber maintained at 4±1°C. For salt treatment, 200 mM NaCl was sprayed on the leaves. Seedlings soaked in liquid MS medium without any treatment were used as control. Whole plants were sampled at 3h and 6h after treatment. For each treatment, six randomly chosen seedlings were pooled together to form a biological replicate. All plant samples were frozen in liquid nitrogen and stored at -80°C until use.

### Expression levels of *MtGRAS* genes under stress treatments

Total RNA was extracted from control and stress-treated samples using Trizol reagent (Invitrogen) based on the manufacturer’s instructions. cDNA was synthesized using approximately 2 μg of RNA according to the manufacturer’s protocol. Real-time quantitative PCR (qRT-PCR) was performed using SYBR Green mix (TaKaRa) on a LightCycler480 Real-Time PCR Detection System (Roche). The fold-change of expression was calculated with *ACTIN* as the internal reference gene. All the primers for qRT-PCR are listed in S1 Table.

## Results

### Identification and structural analysis of *MtGRAS* genes

Using PF03154.11 as a query, we identified 59 *GRAS* genes in *M*. *truncatula*. Most of these genes contained a complete GRAS domain except five (*MtGARS6*, *23*, *25*, *26*, and *49*). To further elucidate the cause and consequences of *MtGRAS* gene expansion, we selected all the genes identified for further analysis, and named them based on their distribution and linear order on the respective chromosomes (Table 1). The peptide length of *MtGRAS* varied greatly ranging from 69 amino acids (*MtGRAS25*) to 1,155 amino acids (*MtGRAS55*). Nearly 52 (88%) *MtGRAS* genes were intronless, which is consistent with most previous studies [1, 12, 58], while three members (*MtGRAS25*, *37*, and *58*) contained just one intron, and four members (*MtGRAS6*, *15*, *23*, and *26*) contained more than one intron (Table 1 and S1 Fig). Molecular weights were significantly different among *MtGRAS* genes, with the smallest at 7 kDa (*MtGRAS25*) and the largest at 129 kDa (*MtGRAS55*). The predicted theoretical pI ranged from 4.72 to 9.8, with a mean of 5.83 (Table 1), which indicates that most of them were weakly acidic.

**Table 1:** Detailed information for 59 *GRAS* genes in the *M*. *truncatula* genome.

Groups	Gene Symbol	Gene ID	ORF(aa)	Number of Exons	Molecular Weight/Da	Theoretical PI
**SHR**	*MtGRAS12*	*Medtr2g089100*	458	1	51691.23	5.58
	*MtGRAS21*	*Medtr2g099110*	452	1	51905.63	5.3
	*MtGRAS27*	*Medtr3g053270*	448	1	50729.91	6.22
	*MtGRAS40*	*Medtr4g095500*	470	1	52854.85	5.78
	*MtGRAS41*	*Medtr4g097080*	504	1	57826.3	5.09
	*MtGRAS47*	*Medtr5g015490*	491	1	55787.56	5.34
	*MtGRAS48*	*Medtr5g015950*	448	1	50729.91	6.22
	*MtGRAS60*	*Medtr8g020840*	554	1	61826.68	5.76
**SCR**	*MtGRAS3*	*Medtr1g069725*	468	1	52996.13	5.81
	*MtGRAS4*	*Medtr1g086970*	480	1	54823.4	6.84
	*MtGRAS8*	*Medtr2g034250*	587	1	67184.17	5.23
	*MtGRAS9*	*Medtr2g034260*	586	1	67296.74	5.17
	*MtGRAS10*	*Medtr2g034280*	577	1	65847.85	5.14
	*MtGRAS37*	*Medtr4g076020*	438	2	48640.81	5.04
	*MtGRAS58*	*Medtr7g074650*	805	2	89030.24	6.1
	*MtGRAS59*	*Medtr7g109580*	567	1	65717.01	5.77
**SCL3**	*MtGRAS6*	*Medtr1g106590*	340	5	38454.02	5.63
	*MtGRAS23*	*Medtr3g022580*	186	3	21812.25	8.27
	*MtGRAS24*	*Medtr3g022830*	438	1	49706.32	6.61
	*MtGRAS25*	*Medtr3g025340*	69	2	7939.17	6.7
	*MtGRAS26*	*Medtr3g027430*	333	5	38417.96	9.51
	*MtGRAS38*	*Medtr4g076140*	472	1	53482.58	6.64
	*MtGRAS46*	*Medtr5g009080*	481	1	54037.85	5.52
**PAT1**	*MtGRAS2*	*Medtr1g029420*	592	1	65944.87	4.79
	*MtGRAS7*	*Medtr2g026250*	598	1	67703.5	5.39
	*MtGRAS11*	*Medtr2g082090*	579	1	64449.5	5.88
	*MtGRAS30*	*Medtr3g089055*	570	1	64226.14	4.93
	*MtGRAS45*	*Medtr4g133660*	554	1	61196.12	5.6
	*MtGRAS51*	*Medtr5g094450*	524	1	59328.15	5.03
	*MtGRAS52*	*Medtr5g097480*	544	1	61006.97	5.96
	*MtGRAS53*	*Medtr6g047750*	624	1	73400.28	6.28
	*MtGRAS55*	*Medtr7g057230*	1155	1	129425.86	8.5
**LISCL**	*MtGRAS13*	*Medtr2g097310*	640	1	73256.09	5.71
	*MtGRAS14*	*Medtr2g097350*	642	1	73409.68	5.4
	*MtGRAS15*	*Medtr2g097380*	563	8	64537.94	7.66
	*MtGRAS16*	*Medtr2g097390*	689	1	78373.19	5.38
	*MtGRAS17*	*Medtr2g097410*	743	1	84140.61	5.42
	*MtGRAS18*	*Medtr2g097463*	657	1	74536.97	5.16
	*MtGRAS19*	*Medtr2g097467*	657	1	74704.13	5.77
	*MtGRAS20*	*Medtr2g097473*	656	1	74496.99	5.63
	*MtGRAS32*	*Medtr4g064120*	628	1	71758.98	5.86
	*MtGRAS33*	*Medtr4g064150*	735	1	83871.84	5.19
	*MtGRAS34*	*Medtr4g064160*	686	1	78061.03	5.72
	*MtGRAS35*	*Medtr4g064180*	628	1	72032.35	5.86
	*MtGRAS36*	*Medtr4g064200*	652	1	73442.76	5.84
**LAS**	*MtGRAS5*	*Medtr1g096030*	445	1	52766.11	5.73
	*MtGRAS39*	*Medtr4g077760*	515	1	60352.59	5.14
**HAM**	*MtGRAS1*	*Medtr0092s0100*	732	1	81808.23	5.56
	*MtGRAS29*	*Medtr3g072710*	508	1	56336.98	5.71
	*MtGRAS31*	*Medtr4g026485*	625	1	70120.89	5.44
	*MtGRAS49*	*Medtr5g019750*	295	2	33956.58	8.61
	*MtGRAS50*	*Medtr5g058860*	506	1	56739.58	4.8
	*MtGRAS57*	*Medtr7g069740*	585	1	67022.34	4.72
	*MtGRAS61*	*Medtr8g077940*	500	1	60492.44	5.58
	*MtGRAS62*	*Medtr8g093070*	507	1	58326.49	4.76
**DELLA**	*MtGRAS28*	*Medtr3g065980*	547	1	60002.63	5.01
	*MtGRAS43*	*Medtr4g104020*	521	1	58794.28	6.43
	*MtGRAS54*	*Medtr7g027190*	674	2	74869.29	5.65
	*MtGRAS63*	*Medtr8g442410*	536	1	60329.3	4.84

### Phylogenetic categories and conserved motif analysis of *MtGRAS* genes

**Fig 1:**
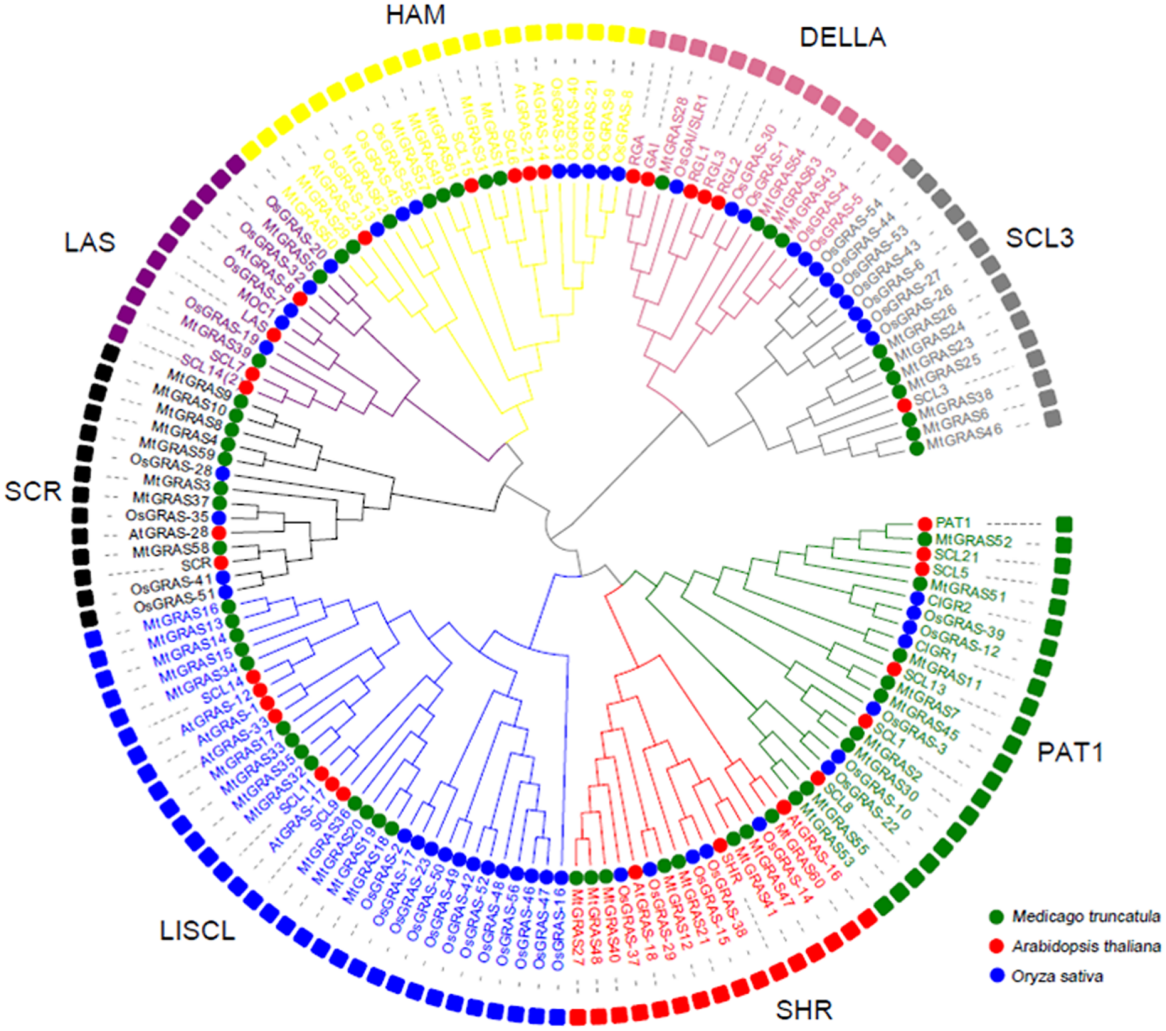
Phylogenetic tree constructed using GRAS proteins from *Medicago truncatula*, *Arabidopsis thaliana*, and *Oryza sativa*. The phylogenetic tree was constructed using MEGA5. Members in the same clade are marked by the same color.

To fully classify the *MtGRAS* gene family, 59 *MtGRAS* genes were analyzed with 32 and 53 *GRAS* genes in *Arabidopsis* and rice, respectively, to construct an unrooted phylogenetic tree using the neighbor-joining (NJ) method in MEGA5. Eight subfamilies were identified based on clade support values, the topology of the phylogenetic tree, and the previous classification of GRAS families in *Arabidopsis* and rice. We identified 9, 13, 8, 8, 7, 8, 4, and 2 *MtGRAS* genes in the PAT1, LISCL, SHR, SCR, SCL3, HAM, DELLA, and LAS sub-branches, respectively (Fig 1).

To explore the potential biological functions of genes in each sub-branch, a detailed comparative analysis was performed (Fig 1). The PAT1 subfamily included nine *MtGRAS* genes including one member (*MtGRAS52*) and two members (*MtGRAS7* and *MtGRAS45*) that have high sequence similarity to *AtPAT1* and *AtSCL13*; *MtGRAS11* was also in the PAT1 subfamily and was the closest homolog of CIGR1 in rice. The LISCL subfamily consists of 13 *MtGRAS* members. Four *MtGRAS* genes (*MtGRAS36*, *20*, *19*, and *18*) share high homology with *AtSCL9*. In addition, another five members (*MtGRAS34*, *15*, *14*, *13*, and *16*) had high similarity with *AtSCL14*, demonstrating that they may function in stress-related processes [59, 60]. The SCR and SHR subfamilies are crucial for stem cell maintenance that occurs during root and shoot development [27]. In our study, two homologous genes (*MtGRAS47* and *MtGRAS41*) of SHR were identified, and one gene (*MtGRAS58*) shared high similarity with SCR. *SCL3* regulates root cell elongation by integrating multiple signals in *Arabidopsis* [28]. Seven *MtGRAS* genes (*MtGRAS26*, *24*, *23*, *25*, *38*, *6* and *46*) belonged to the SCL3 sub-branch, implying a similar function in root development. *MtGRAS29*, which belonged to the HAM subgroup, has been suggested to participate in nodule morphogenesis [2]. *MtGRAS50*, the closest paralog of *MtGRAS29*, may function in the same pathway as well. In the DELLA subfamily, four members (*MtGRAS54*, *63*, *43*, and *28*) had the highest similarity to *RGA* and *GAI*. Two *MtGRAS* members (*MtGRAS5* and *39*) were identified as part of the LAS subgroup, which has several genes that have been found to regulate meristem formation [29–31].

**Fig 2:**
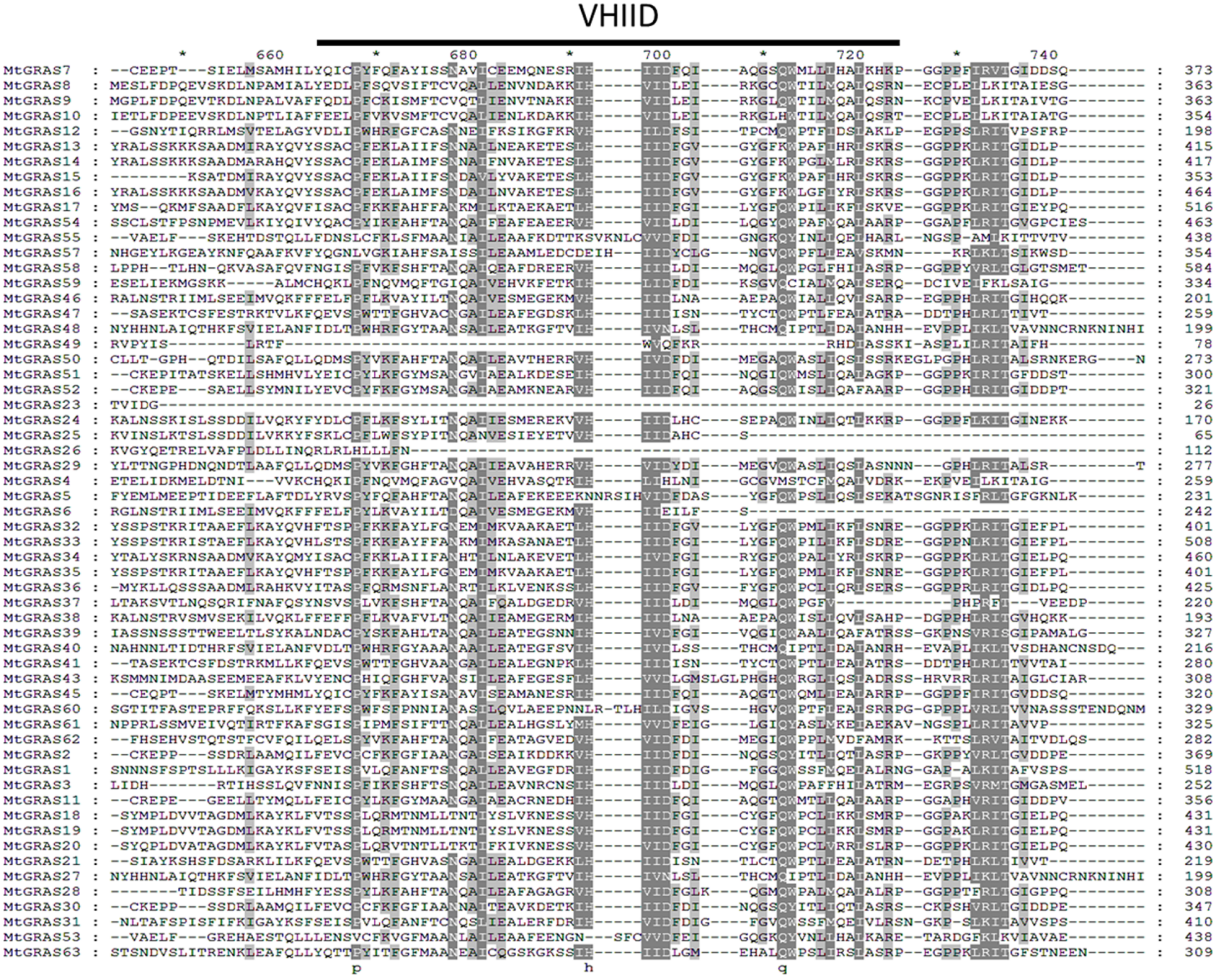
Multiple sequence alignment of 59 *MtGRAS* genes by ClustalW. The most conserved motif of VHIID is underlined with a black solid line.

**Fig 3:**
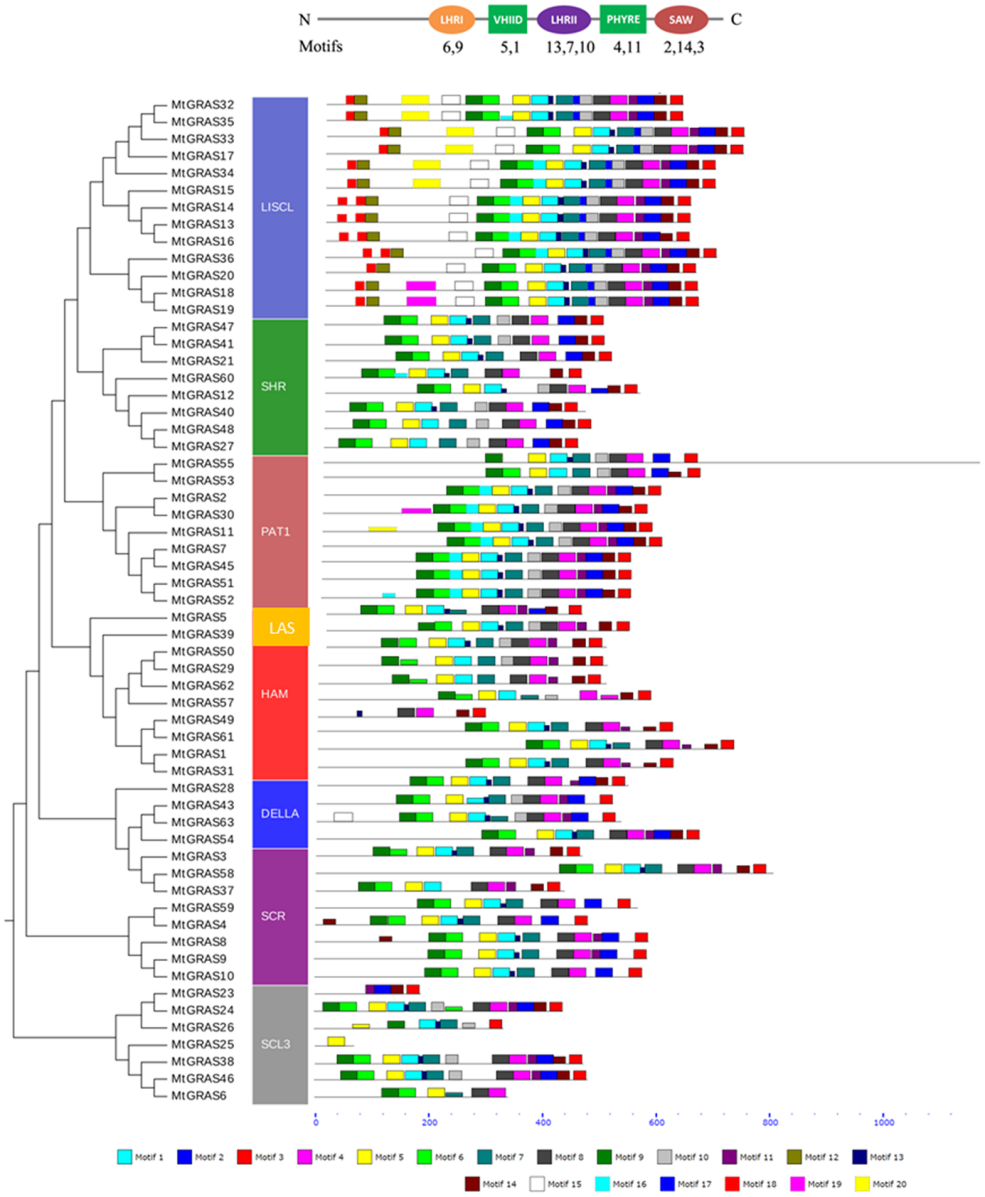
The distribution of conserved motifs in MtGRAS proteins. Neighbor-joining tree of MtGRAS proteins is shown on the left. MtGRAS proteins are categorized into eight distinct clusters including LISCL, SHR, PAT1, LAS, HAM, DELLA, SCR, and SCL3, and are represented by different colored vertical boxes. The horizontal colored boxes indicate conserved motifs within each protein. Conserved domains and corresponding motifs are shown at the top. A scale of protein length is shown at the bottom.

Using multiple sequence alignment, the characteristic conserved domains located in the C-termini were identified including VHIID, LHRI, LHRII, PFYRE, and SAW (Fig 2 and S2–S5 Figs). We further explored conserved motifs in *MtGRAS* using MEME tools [51]. In total, 20 conserved motifs were found, and most of them had a similar distribution within the same subgroup (Fig 3). The logo of these motifs is listed in S6 Fig. The motifs located in the GRAS domain, including LHRI (motif6, motif9), VHIID (motif5, motf1), LHRII (motif13, motif7, motif10), PFYRE (motif4, motif11), and SAW (motif2, motif14, motif3), were shared across almost all *MtGRAS* members. In addition, motif8 was situated between LHRII and PFYRE and conserved among most *MtGRAS* subfamilies, suggesting its functional importance. Other motifs located outside the GRAS domain regions showed subgroup specific patterns. Motif16 was located between LHR1 and VHIID, and was specific to the PAT1 and LISCL subgroups, while motif17 was nested within LHRII and was LISCL specific. Motif12 and motif18 were located in the N-termini and were also only present in the LISCL subgroup (Fig 3).

### Chromosomal distributions and duplication analysis of *MtGRAS* genes

**Fig 4:**
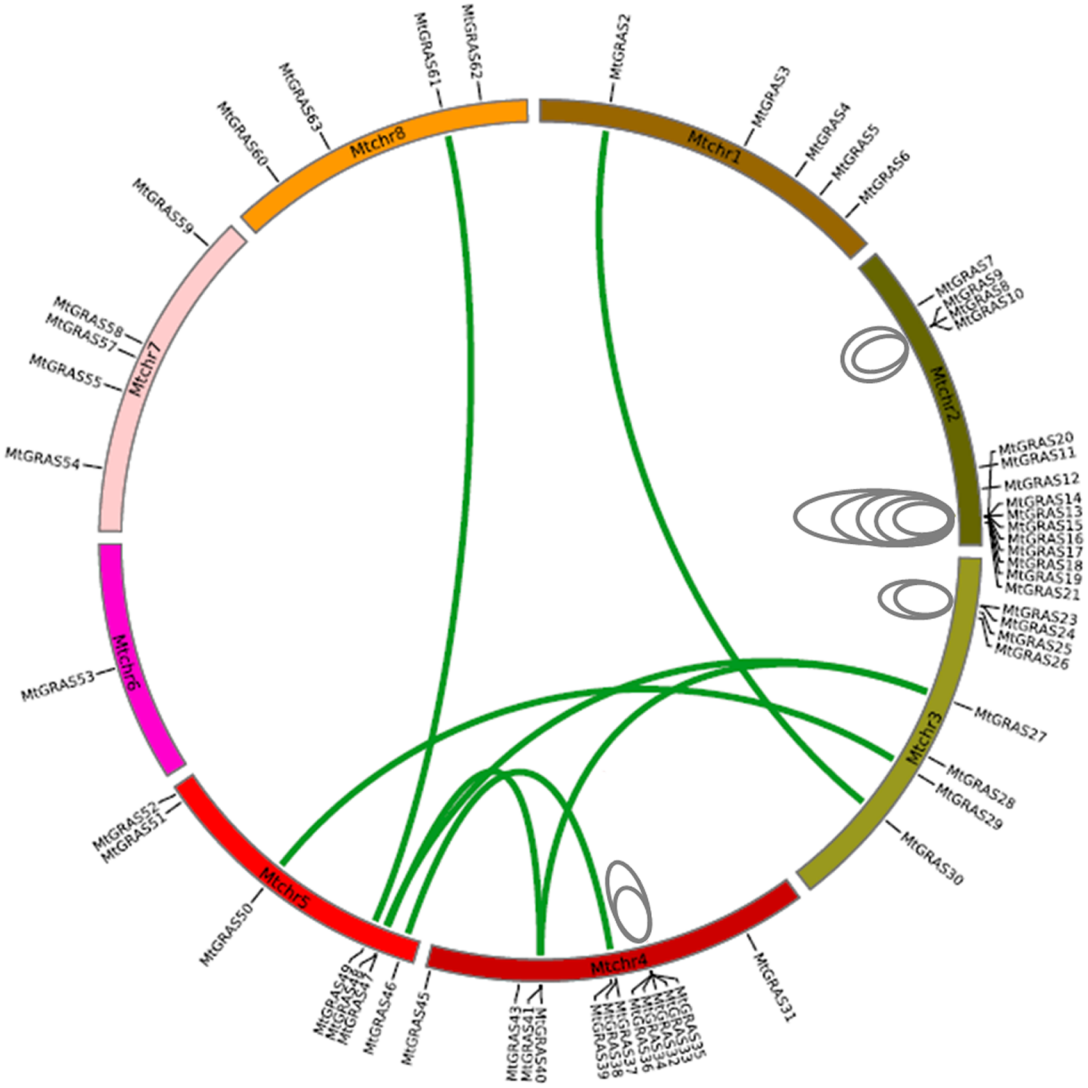
Chromosomal distribution and expansion analysis of *MtGRAS* genes in *M*. *truncatula*. Green lines show paralogous genes duplicated by segmental duplication (SD), and grey lines indicate tandem duplication (TD).

Fifty-nine *MtGRAS* genes were mapped to the chromosomes of *M*. *truncatula*; *MtGRAS1* was excluded because it was positioned on a scaffold (Table 1). The distribution of *MtGRAS* genes among the chromosomes was uneven. Chr2 and chr4 are the “hot regions”, and contained 15 (25.9%) and 13 (22.4%) *MtGRAS* genes, respectively; chr6 is the “cold region”, and contained only one (1.7%) *MtGRAS* gene. Moreover, 5, 8, 7, 5, and 4 *MtGRAS* genes were found on chr1, chr3, chr5, chr7, and chr8, respectively (Fig 4). Based on these distributions, we explored duplication events of *MtGRAS* genes. Seventeen duplicated *MtGRAS* gene pairs were identified. Most duplication events occurred in chr2 (n = 7), chr3 (n = 6), and chr4 (n = 5); one duplication event occurred in chr1, chr5, and chr8; and no duplication events were identified in chr6 and chr7 (Fig 4). We also verified the types of duplication. The results suggested that 11 duplicated gene pairs arose from tandem duplications, while six pairs were segmental duplications (Table 2).

**Table 2:** Detailed information of duplicated *MtGRAS* genes.

Groups	Gene symbol	Homolog	Protein identity	Types of duplication	Ka^c^	Ks^d^	Ka/Ks
PAT1	*MtGRAS2*	*MtGRAS30*	76.21%	SD^a^	0.149	0.819	0.181
LISCL	*MtGRAS14*	*MtGRAS16*	84.12%	TD^b^	0.081	0.092	0.882
LISCL	*MtGRAS15*	*MtGRAS16*	76.21%	TD	0.177	0.292	0.607
LISCL	*MtGRAS13*	*MtGRAS16*	85.85%	TD	0.070	0.099	0.708
LISCL	*MtGRAS19*	*MtGRAS18*	71.40%	TD	0.106	0.261	0.406
LISCL	*MtGRAS19*	*MtGRAS20*	91.62%	TD	0.040	0.085	0.467
LISCL	*MtGRAS32*	*MtGRAS35*	97.77%	TD	0.010	0.047	0.218
LISCL	*MtGRAS32*	*MtGRAS33*	78.15%	TD	0.121	0.404	0.300
SHR	*MtGRAS27*	*MtGRAS40*	69.18%	SD	0.206	1.115	0.185
SHR	*MtGRAS41*	*MtGRAS47*	74.01%	SD	0.169	1.048	0.161
HAM	*MtGRAS49*	*MtGRAS61*	60.54%	SD	0.315	1.407	0.224
HAM	*MtGRAS29*	*MtGRAS50*	66.67%	SD	0.248	1.543	0.161
SCL3	*MtGRAS24*	*MtGRAS23*	78.38%	TD	0.122	0.333	0.367
SCL3	*MtGRAS24*	*MtGRAS25*	68.12%	TD	0.221	0.364	0.607
SCL3	*MtGRAS38*	*MtGRAS46*	77.66%	SD	0.134	0.968	0.139
SCR	*MtGRAS9*	*MtGRAS8*	83.89%	TD	0.082	0.267	0.307
SCR	*MtGRAS9*	*MtGRAS10*	82.24%	TD	0.093	0.284	0.326

^a^ Segmental duplication.

^b^ tandem duplication.

^c^Non-synonymous substitution rate.

^d^Synonymous substitution rate.

To understand the evolutionary process of gene duplications, we evaluated the positions of duplicated genes. Seven duplicated *MtGRAS* genes (*MtGRAS9*, *14*, *15*, *13*, *19*, *24*, *2*, and *49*) were positioned near the telomeres of each chromosome, and three genes (*MtGRAS24*, *29*, and *38*) were located around the centromeres, implying that the highly repetitive nature of these regions may lead to gene duplication (Fig 4). To reveal the evolutionary dynamics of duplicated *MtGRAS* genes, we calculated nonsynonymous substitution rates (Ka) and synonymous substitution rates (Ks) between duplicated genes. All of the duplicated gene pairs have Ka/Ks values <1, suggesting that purifying selection acted on them (Table 2).

### Expression pattern analysis of *MtGRAS* genes in different tissues

*GRAS* transcription factors have crucial roles in various biological pathways. In this study, we analyzed the expression profiles in different tissues including root, blade, flower, bud, and nodule using publicly available Illumina RNA-seq data. Based on the transcriptome analysis, 53,777 expressed genes were identified, including: 52,236 in blade; 51,236 in bud; 54,032 in flower; 55,041 in nodule; and 52,437 in root.

**Fig 5:**
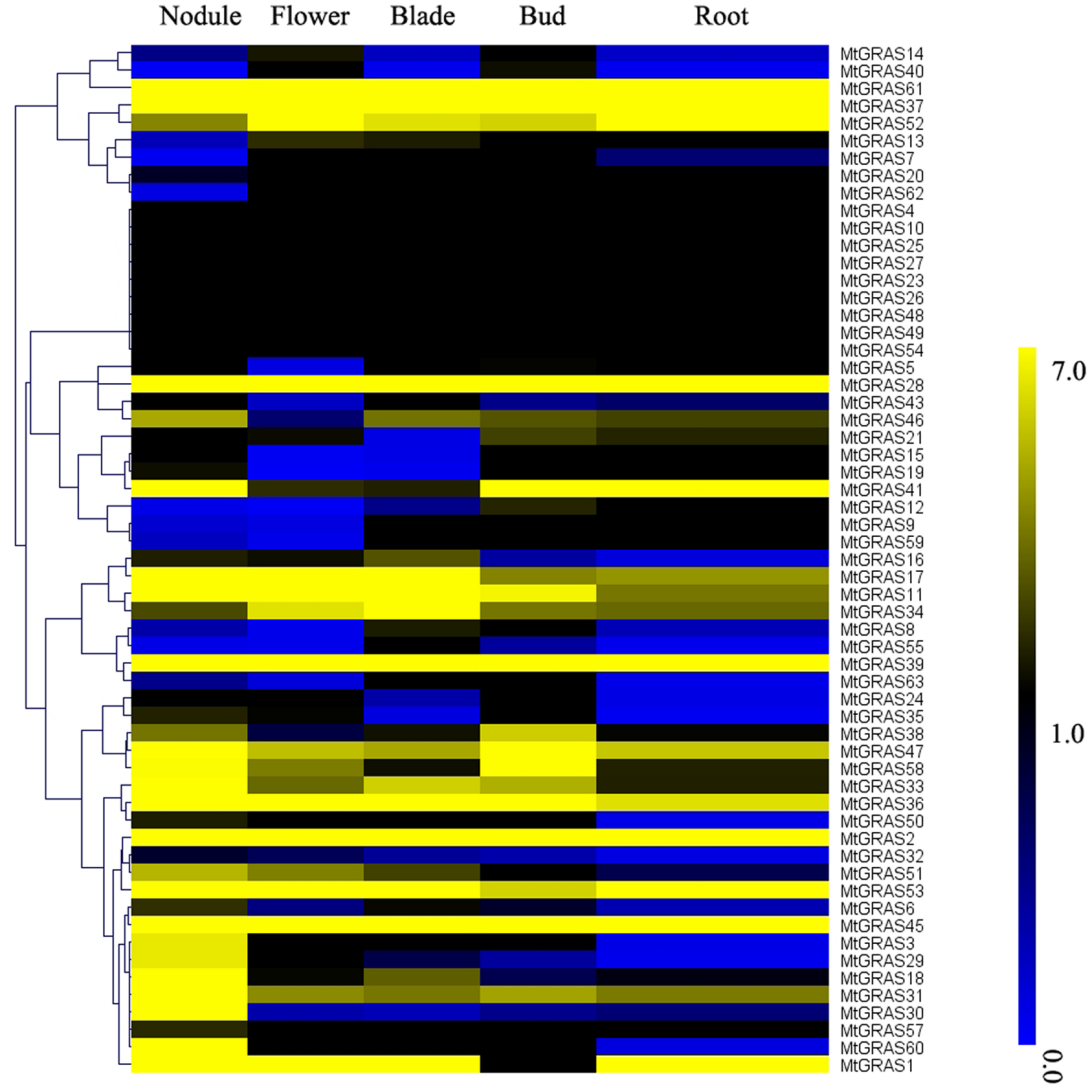
Expression profile cluster analysis of *MtGRAS* genes.

**Fig 6:**
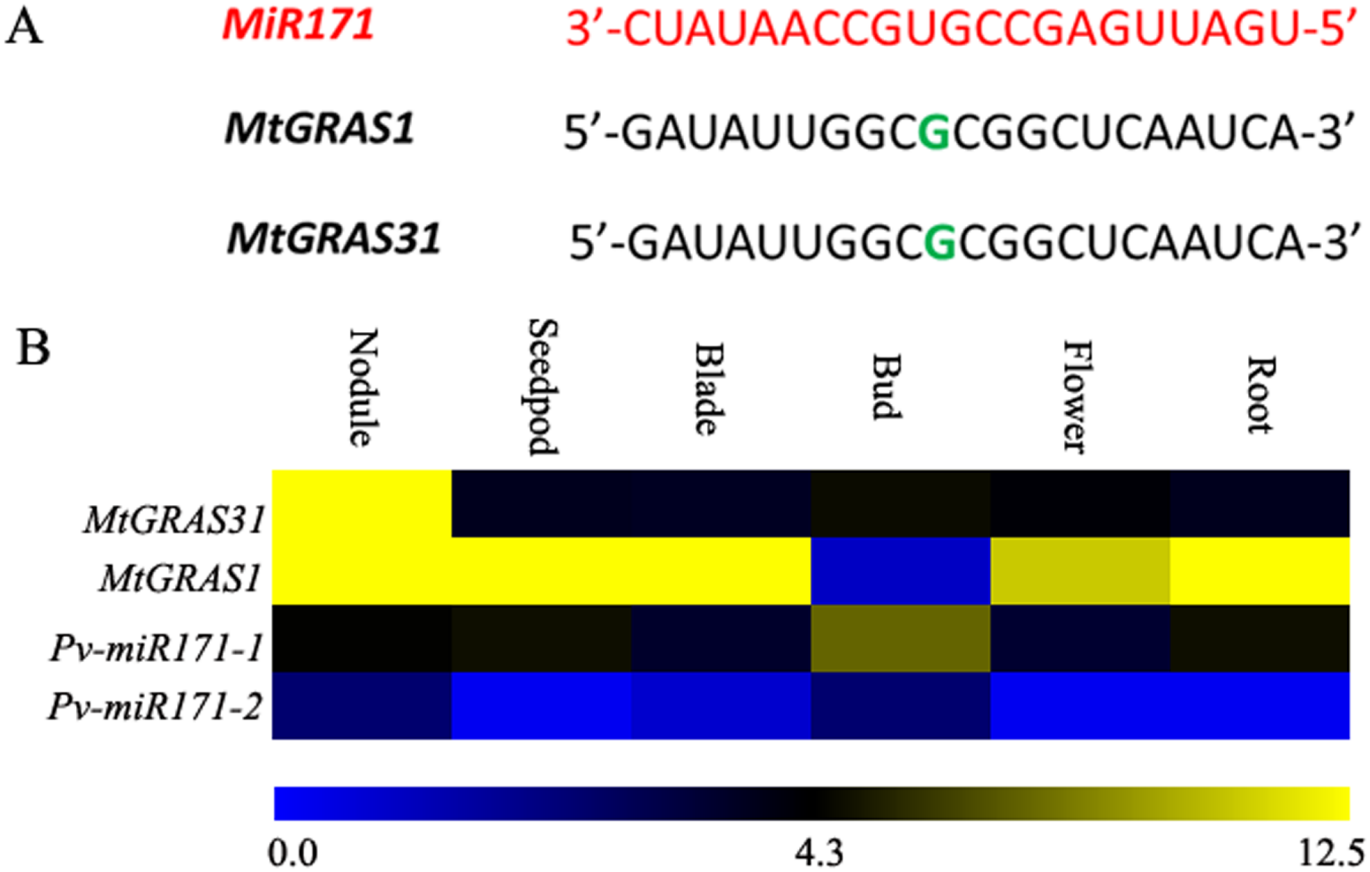
Putative *Pv-miR171* genes and their target *MtGRAS* genes. (A) *miRNA171* cleavage sites in *MtGRAS* genes. (B) The expression patterns of *Pv-miR171* genes and their target *MtGRAS* genes in different tissues. *Pv-miR171-1*: Medtr4g111710; *Pv-miR171-2*: Medtr1g099290.

In our data, expression of most *MtGRAS* genes was identified in at least in one tissue. Expression of nine *MtGRAS* genes (*MtGRAS4*, *10*, *23*, *25*, *26*, *27*, *48*, *49* and *54*) was not detected in our transcriptome data, which may be the result of spatial and temporal expression patterns or uncharacterized pseudogenes. The FPKM values of each *MtGRAS* are shown in S2 Table and the expression profiles were clustered across six tissues (Fig 5). Among the 50 expressed *MtGRAS* genes, 38 were highly expressed (FPKM >1), while the other 12 had low expression (FPKM <1). Interestingly, the FPKM values of six genes (*MtGRAS1*, *28*, *30*, *45*, *53*, and *61*) were higher than 40, indicating their important role in the development of *M*. *truncatula*. We also compared expression levels among different tissues. Interestingly, 30 *MtGRAS* genes had the highest expression in nodules, and 5, 7, 4, and 4 *MtGRAS* genes had the highest expression in blade, bud, flower, and root, respectively (S2 Table). The high proportion of *MtGRAS* genes expressed in nodules indicates that additional *MtGRAS* genes besides *NSP1* and *NSP2* participate in the process of nodulation. Furthermore, three *Arabidopsis GRAS* genes (*SCL6*, *22*, and *27*) in the HAM subfamily are post-transcriptionally regulated by *miR171*. Here, the two closest homologs of *SCL6*, *MtGRAS1* and *MtGRAS31*, were identified as having a putative binding site for *miR171* (Fig 6A). Two *Pv-miR171* genes were detected in the *Medicago* genome. The expression pattern of *Pv-miR171* genes were negatively correlated with their targets. Both *MtGRAS1* and *31* exhibited highest expression in nodules, while *Pv-miR171* genes (*Pv-miR171-1* and *Pv-miR171-2*) showed the lowest expression in this tissue. In buds, *MtGRAS1* and *31* accumulated the least transcript, but the transcript of *Pv-miR171* genes, especially *Pv-miR171-1*, was the highest among different tissues(Fig 6B).

**Fig 7:**
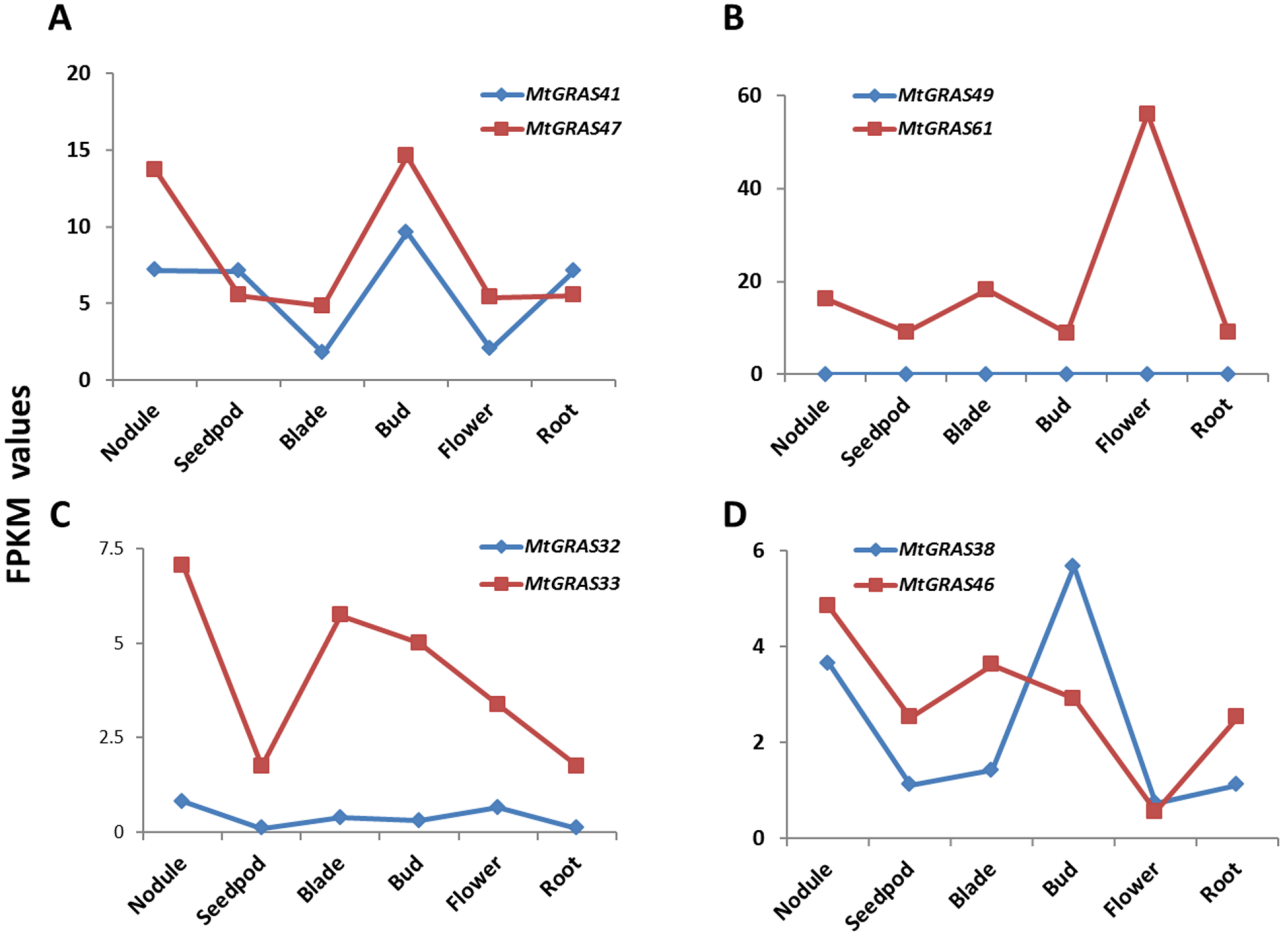
Comparative analysis of expression profiles of duplicated *MtGRAS* genes. The X-axis represents different tissues of *M*. *truncatula*. The Y-axis shows the expression values (FPKM) obtained using RNA-seq data.

Since duplicated genes can exhibit significant variation in gene expression, we next explored the expression divergence of 17 sets of *MtGRAS* duplicated genes. Detailed expression information of these genes is listed in S3 Table. Eleven duplicated gene pairs shared similar expression patterns with different expression levels. For example, both *MtGRAS47* and its duplicated gene *MtGRAS41*, had higher expression in bud and nodule, but showed lower expression in blade and flower (Fig 7A). This pattern was also observed in gene pairs *MtGRAS2/30*, *19/18*, *19/20*, *29/50*, *9/8*, *9/10*, *14/16*, *15/16*, *13/16* and *32/35* (S7 Fig). After the duplications, four genes were not expressed in our transcriptome data, including *MtGRAS49* in the gene pair *49/61*, *27* in *27/40*, *23* in *24/23*, and *25* in *24/25* (Fig 7B and S7 Fig). For example, *MtGRAS61* was expressed in six tissues, and had the highest expression in flower, but the transcript level of the duplicated gene *MtGRAS49* was not detected (Fig 7B). Intriguingly, two duplicate gene pairs, *MtGRAS32/33* and *38/46* –showed different expression profiles (Fig 7C and 7D). In the gene pair, *MtGRAS32*/*33*, *MtGRAS32* had higher expression in nodule and blade, whereas *MtGRAS33* had higher expression in nodule and flower. *MtGRAS38* was highly expressed in bud, whereas the transcripts of its homologous gene *MtGRAS46* were enriched in nodules. In all, the expression differences of duplicated genes presented here implies that genes may have various evolutionary outcomes after duplication.

### Responses of *MtGRAS* genes to different stress treatments

**Fig 8:**
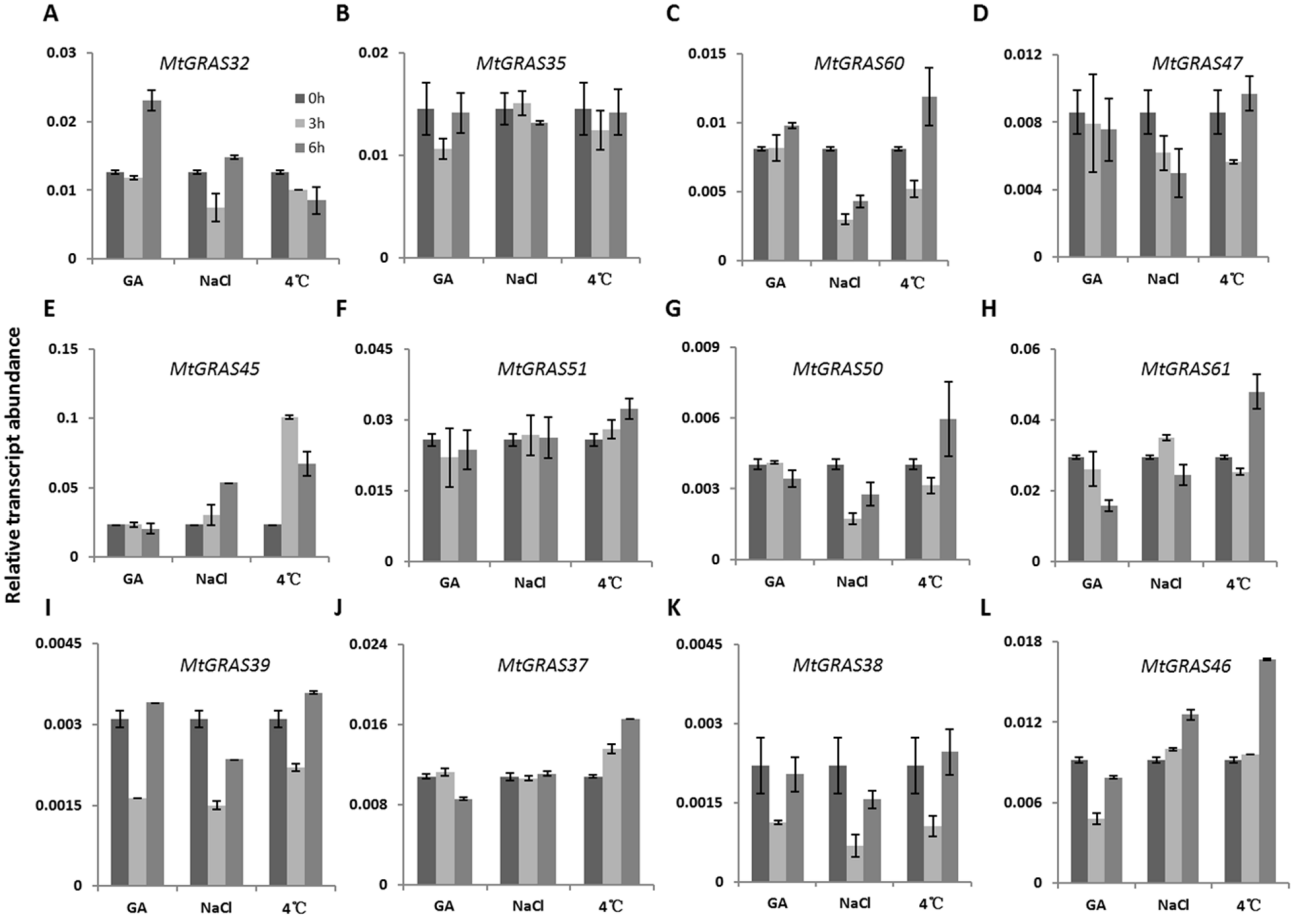
Relative expression levels of *MtGRAS* genes in different abiotic stress treatments. Three-week-old *M*. *truncatula* (cv. Jemolong A17) seedlings were subjected to various stress treatments including GA3 gibberellin (30 μM), cold (4°C), and salt (200 mM). The expression level was measured at 0h, 3hm and 6h after treatment. Error bars show the standard error of three replicates.

We further examined changes in transcript abundance in response to stress treatments including GA3, salt, and cold using qPCR. Twelve *MtGRAS* genes were used to assess responses to treatments, including: *MtGRAS32* and *35* (LISCL subfamily); *60* and *47* (SHR subfamily); *45* and *51* (PAT1 subfamily); *50* and *61* (HAM subfamily); *37* (SCR subfamily); 39 (LAS subfamily); and *38* and *46* (SCL3 subfamily). All of these genes exhibited differential expression in response to at least one stress treatment (Fig 8). After GA3 treatment, the expression levels of most *MtGRAS* genes (10) were downregulated. Five genes (*MtGRAS35*, *51*, *39*, *38*, and *46*) had decreased expression at 3h and recovered, to some extent, at 6h. The rest of the genes (*MtGRAS47*, *45*, *50*, *61*, and *37*) had decreased transcripts after GA3 treatment, and reached the lowest expression level at 6h. Only two genes, *MtGRAS32* and *MtGRAS60*, were positively upregulated and reached the highest expression level at 6h. Under salt treatment, the transcripts of *MtGRAS51* and *37* were not changed compared to the control, which indicated that they might not participate in the response to salt stress during the development of *M*. *truncatula*. In the remaining 10 *MtGRAS* genes, three genes (*MtGRAS45*, 46, and *32*) were upregulated, and reached the highest expression level at 6h; the other seven genes were clearly downregulated, including four genes (*MtGRAS60*, *50*, *39*, and *38*) that had the lowest expression at 6h, and three genes (*MtGRAS35*, *47*, and *61*) with the lowest expression at 3h. In the cold stress treatment, the transcripts of most *MtGRAS* genes were upregulated, except in two genes (*MtGRAS32* and *35*). The expression levels of six genes (*MtGRAS60*, *47*, *50*, *61*, *39*, and *38*) decreased at 3h but increased at 6h, while three genes (*MtGRAS51*, *MtGRAS37*, and *MtGRAS46*) increased in linear order and reached the highest expression level at 6h. Finally, the expression of *MtGRAS45* was highest at 3h but decreased at 6h (Fig 8).

## Discussion

*GRAS* transcription factors play essential roles in regulating plant growth and development. However, the prevalence and functional diversity of the *GRAS* family in *M*. *truncatula* have not been thoroughly investigated. In this study, we performed a comprehensive analysis of the *GRAS* gene family in *M*. *truncatula*. The features of *MtGRAS* genes, including their chromosomal distribution, phylogenetic classification, expression profiles, and responses to various stresses were explored. Results of these analyses allowed us to study the evolution of the *GRAS* family and draw hypotheses about the potential functions of unidentified genes.

Our results demonstrated that duplication was the evolutionary force behind *GRAS* gene family expansion. First, the number of *GRAS* was different among species. In this study, we identified 59 *GRAS* genes in *M*. *truncatula*, which is lower than the number in *Populus* (106) [15], but higher than in other species such as *Arabidopsis* (33) [1], rice (57) [1], Chinese cabbage (46) [9], tomato (53) [12] and grapevine (52) [10]. Gene duplication might cause these differences in numbers of *GRAS* family members. Second, *MtGRAS* genes were unevenly distributed among the chromosomes, with the “hot regions” on chr2 (15 members) and chr4 (13 members), and the “cold region” on chr6 (1 member). Interestingly, more duplication events were found in the hot regions (7/chr2, 4/chr4) (Fig 4). We further related the duplication events to chromosome positions, and found that duplicated *MtGRAS* gene pairs tend to be located in regions with low levels of conservation (10 members), such as peritelomeres and pericentromeres; tandem and segmental duplication were enriched in these regions because of the composition of repetitive elements (Fig 4). Furthermore, among the 17 duplicated *MtGRAS* gene pairs, seven belonged to the LISCL subfamily and clustered in chr2 and chr4, suggesting that gene duplication might cause proliferation of the LISCL subfamily. Previous studies have found that duplication is common in the *GRAS* gene family. For example, 2/34, 15/53, 17/60, and 40/106 *GRAS* genes were identified as duplicated genes in *Arabidopsis* [1], tomato [12], rice [1], and *Populus* [15], respectively, which further validates the contribution of duplication to expansion of the *GRAS* gene family. Third, nearly 88% of *MtGRAS* genes were intronless, consistent with other species such as tomato (77.4%) [12], *Prunus mume* (82.2%) [58], and *Arabidopsis* (67.6%) [1]. Intronless genes have also been discovered in other large gene families, such as DEAD box RNA helicase [61] and F-box transcription factors [62]. Rapid duplication after horizontal gene transfer from bacteria is the main contributor to the high proportion of intronless genes.

To our knowledge, transcription factors belonging to the same taxonomic clade exhibit recent evolutionary origins and specific conserved motifs associated with functional specification. Because of this, a comparison of homologous genes in the *MtGRAS* family, including protein sequences and expression profiles, would be an effective method to predict the function of uncharacterized genes. In this study, 50 *MtGRAS* genes were expressed in at least one tissue according to our transcription analysis, and the expression patterns varied across a variety of tissues, as previously reported in *Populus*, *Prunus mume*, tomato, and grapevine. Noticeably, more than half (30/59) of the *MtGRAS* genes had the highest expression in the nodule, and 5, 7, 4, and 4 members were preferentially expressed in blade, bud, flower, and root, respectively. This result suggested that the functions of *MtGRAS* have dramatically diverged. Genes belonging to the LAS subfamily have been found to participate in regulating axillary meristem development. For example, the mutation of *MOC1* resulted in the phenotype of no tillers except for a main culm in rice [29]. In an *Arabidopsis* knockout, the homolog of *MOC1*, named *AtLAS*, led to an inability to form lateral shoots during vegetative development [31]. In *M*. *truncatula*, two *MtGRAS* genes (*MtGRAS5* and *MtGRAS39*) belonged to the LAS subgroup. Interestingly, both of them were expressed highest in bud, which indicated that they might also play a vital role in axillary meristem formation (Fig 5 and S2 Table). The *DLT* gene in rice and its orthologs, *AtSCL28* in *Arabidopsis* and *VviGRAS8a* in *Vinus vinfera*, modulate the expression of a brassinosteroid-related gene [59, 63]. In tomato, the ortholog of *DLT* was validated to be involved in the flower-fruit transition by mediating brassinosteroid signaling [12]. In our study, *MtGRAS12* was the homolog of *DLT*, and was preferentially highly expressed in bud, but had low expression in flower (Fig 5 and S2 Table). The result suggested that *MtGRAS12* might function in response to brassinosteroid signaling during bud development. DELLA genes participate in various developmental processes including flower development, stem elongation, and seed germination [28]. In addition, the DELLA proteins also participate in hormone signaling pathways, such as the gibberellin, jasmonate, auxin, brassinosteroid, and ethylene pathways [64]. In our data, the closest homolog of RGA and GAI, *MtGRAS28*, was highly expressed in different tissues (FPKM>40) including root, seedpod, and blade, supporting a role in diverse developmental processes (Fig 5 and S2 Table).

Generally, the evolutionary fate of duplicated genes includes nonfunctionalization, neofunctionalization, or subfunctionalization [65, 66]. We further evaluated the evolutionary dynamics and consequences of duplicated *MtGRAS* genes. All of the duplicated genes were under purifying selection (Ka/Ks <1), implying that these genes were still strongly controlled after duplication (Table 2). We next examined the divergence of expression in 17 sets of duplicated *MtGRAS* gene pairs. Eleven duplicated genes showed similar expression patterns to the original gene, but with different expression levels; four duplicated genes were not expressed in our transcription dataset (Fig 7 and S3 Fig). Furthermore, the duplicated gene pairs *MtGRAS38/46* and *MtGRAS32/33* exhibited different expression patterns, suggesting that novel functions might evolve after duplication (Fig 7). Further efforts need to be made to elucidate the functional diversity of duplicated genes.

Previous studies have demonstrated that the GRAS protein could interact with ERN to regulate gene expression during rhizobial infection [67]. Two *MtGRAS* genes, *NSP1* and *NSP2*, belonging to SHR and HAM, respectively, were associated with enhancing Nod factor elicitation [2]. In our study, more than half (30/50) of the *MtGRAS* genes had the highest expression in nodules, which implied that *MtGRAS* genes other than *NSP1* and *2* might participate in the process of nodulation. In *Arabidopsis*, three *GRAS* members in the HAM subfamily were post-transcriptionally regulated by *miR171* (*AtSCL6*, *22*, and *27*). Interestingly, in the present study, the two closest homologs of *AtSCL6* (*MtGRAS1* and *31*) were found to have a putative binding site for *miR171*. Both of these genes were highly expressed in nodules, especially the *MtGRAS1* gene, which had an FPKM value higher than 40 (Fig 6). These results indicated that *mi172* might also be involved in nodule development of legumes [37–39].

Numerous studies have found that transcription factors in the *GRAS* family could be influenced by various biotic and abiotic stresses. Gibberellin, auxin, brassinosteroid, abscisic acid, ethylene, and salicylic acid also play important roles in a diverse array of developmental processes including germination, flowering time, and stem elongation [64]. GAI, RGA, and RGL in the DELLA subfamily were repressors of gibberellin signaling [4–6]. Loss of function in the *Arabidopsis* mutants *scr* and *shr*, resulted in hypersensitization to abscisic acid [6]. *BnSCL1* in *Brassica napus* showed differential dose responses to auxin in shoots and roots [68]. Recently, a study in tomato demonstrated that the expression level of *GRAS* genes could be modulated by signaling of multiple phytohormones, including gibberellin, auxin, brassinosteroid, ethylene, and salicylic acid [12]. Additionally, several studies have revealed the participation of *GRAS* genes in response to abiotic stresses, such as cold, drought, salt, and heat. In *Arabidopsis*, over-expression of a poplar *GRAS* gene, *PtSCL7*, enhanced tolerance to salt and drought stress [69]. *SCL14* in *Arabidopsis* was involved in the activation of a broad-spectrum detoxification network, and its ortholog in rice, *OsGRAS23*, was involved in regulating the drought stress response [23, 59, 60]. The gene *BoGRAS* was significantly upregulated during heat stress in *Brassica oleracea* [70]. In our study, 12 *MtGRAS* genes from different subgroups were randomly selected to explore their responses to biotic (GA3) and abiotic (NaCl and 4°C) stresses. We found that nearly all *MtGRAS* genes could be affected by different stress treatments (Fig 8). Most *MtGRAS* genes (10/12) were downregulated after treatment with GA3, while only two genes were upregulated, implying that most *MtGRAS* genes had negative roles in response to this hormone. Under salt treatment, the expression levels of seven genes decreased, and the expression levels of three genes increased, suggesting that *MtGRAS* genes modulate the signaling of response to salt through complicated networks. The majority of genes (10/12) increased their expression level under the 4°C treatment, indicating they might positively regulate the response gene in the cold condition. In addition, most *MtGRAS* genes could be influenced by both hormone and abiotic stress treatments, indicating the coordinated response of these two environmental determinants.

## Conclusions

In this study, we performed a genome-wide analysis of the *GRAS* gene family in *M*. *truncatula* based on publicly available genome data. Fifty-nine *MtGRAS* genes were identified and categorized into eight subfamilies by phylogenetic analysis. Conserved motif analysis combined with expression profile measurement in different tissues and environmental treatments demonstrated the functional conservation and diversity of *MtGRAS* genes. The evolutionary dynamics of *MtGRAS* family members was further inferred by analyzing the cause and consequence of duplicated *MtGRAS* gene pairs. We foresee that these results will be of great value for further functional characterization of the *MtGRAS* gene family and for genetic improvements in agronomic traits or stress tolerance in legumes.

## Supporting information

S1 Fig Gene structure of the *MtGRAS* genes. Pink boxes represent exons, blue arrows represent UTRs, and black lines show introns. The lengths of the exons, introns and UTRs were drawn to scale.
(TIF)

S2 Fig The conserved domain of LHRI was underlined by multiple sequence alignment of 59 *MtGRAS* genes. (TIF)

S3 Fig The conserved domain of LHRII was underlined by multiple sequence alignment of 59 *MtGRAS* genes. (TIF)

S4 Fig The conserved domain of PFYRE was underlined by multiple sequence alignment of 59 *MtGRAS* genes. (TIF)

S5 Fig The conserved domain of SAW was underlined by multiple sequence alignment of 59 *MtGRAS* genes. (TIF)

S6 Fig Conserved motifs detected in MtGRAS proteins using MEME software. The logo represents conserved amino acids sequences in different motifs, and the heights of letters in the logo represent the frequency of amino acid at specific positions.
(TIF)

S7 Fig The comparative analysis of expression profiles of duplicated *MtGRAS* genes. X-axis represents different tissues of *M*. *truncatula*. Y-axis shows the expression values (RPKM) obtained using RNA-seq data.
(TIF)

S1 Table The primers used in RT-PCR experiments. (DOCX)

S2 Table Expression levels of *MtGRAS* genes measured by transcriptome analysis. (DOCX)

S3 Table Expression pattern of duplicated *MtGRAS* genes. (DOCX)
