# Supplementary figures and images for: Genome-wide characterization of GRAS family genes in Medicago truncatula reveals their evolutionary dynamics and functional diversification
Source: PLoS One. 2017 Sep 25;12(9):e0185439. doi: 10.1371/journal.pone.0185439 (PMC5612761; doi:10.1371/journal.pone.0185439)

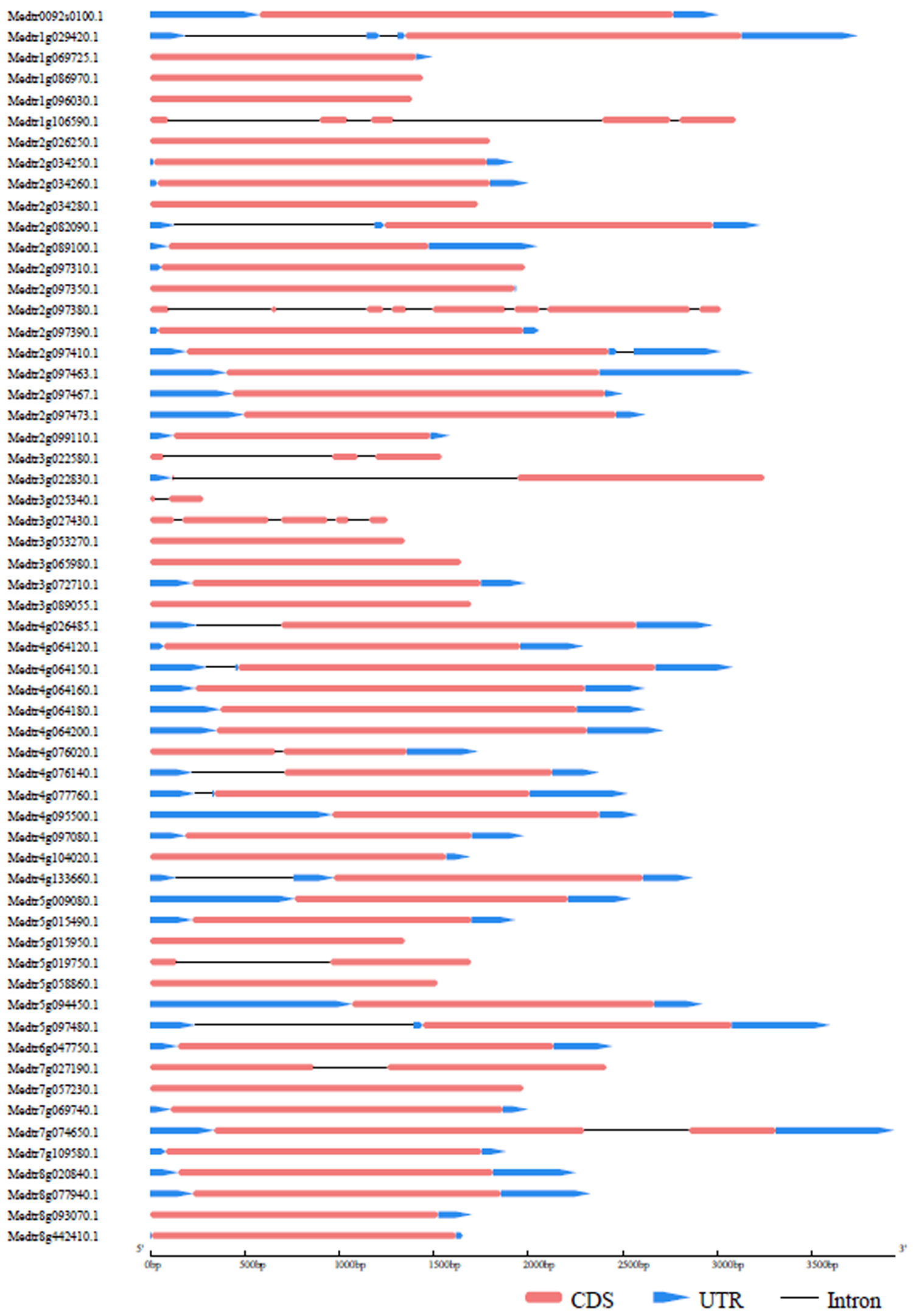

Supplement: S1 Fig — Pink boxes represent exons, blue arrows represent UTRs, and black lines show introns. The lengths of the exons, introns and UTRs were drawn to scale. (TIF) [file pone.0185439.s001.tif]

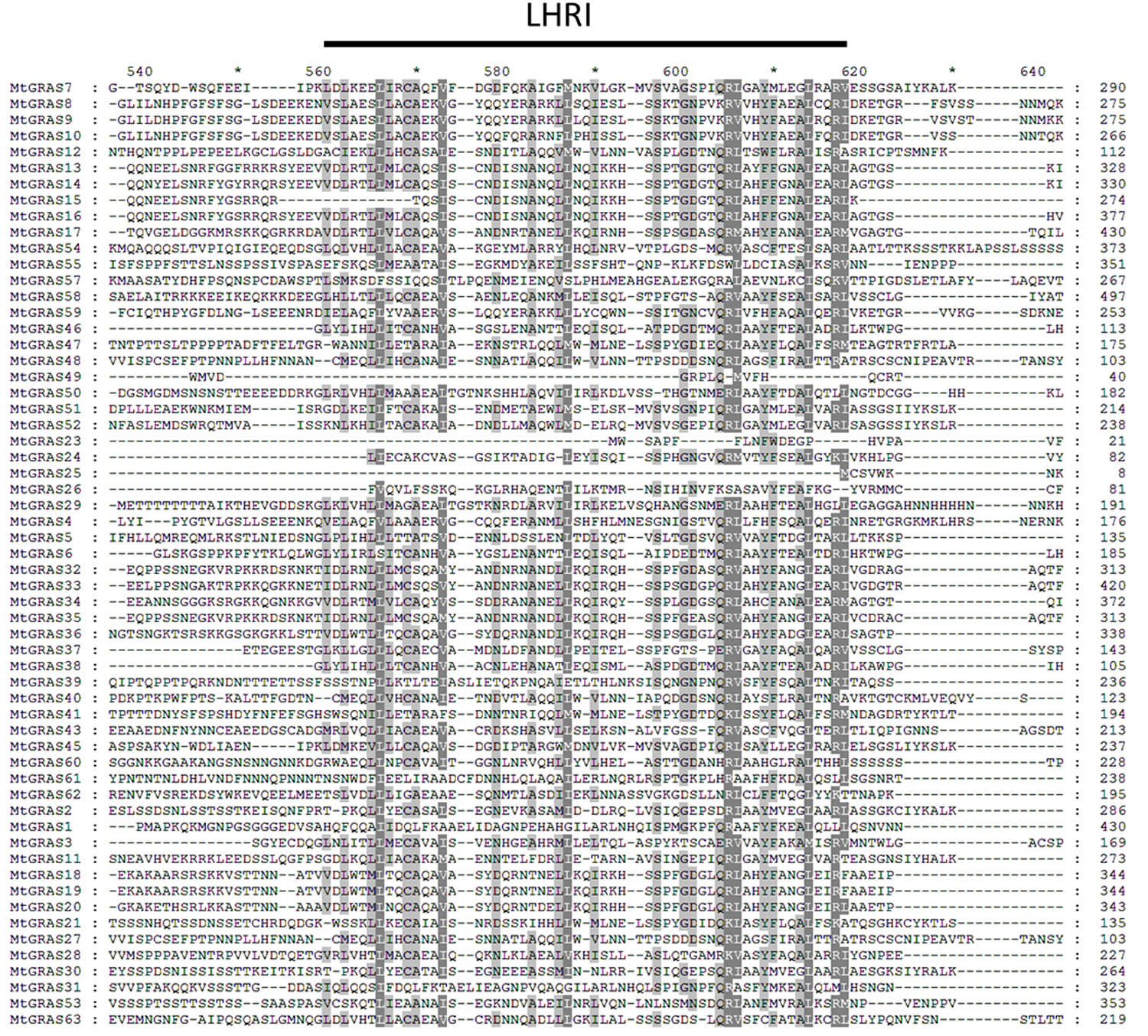

Supplement: S2 Fig — (TIF) [file pone.0185439.s002.tif]

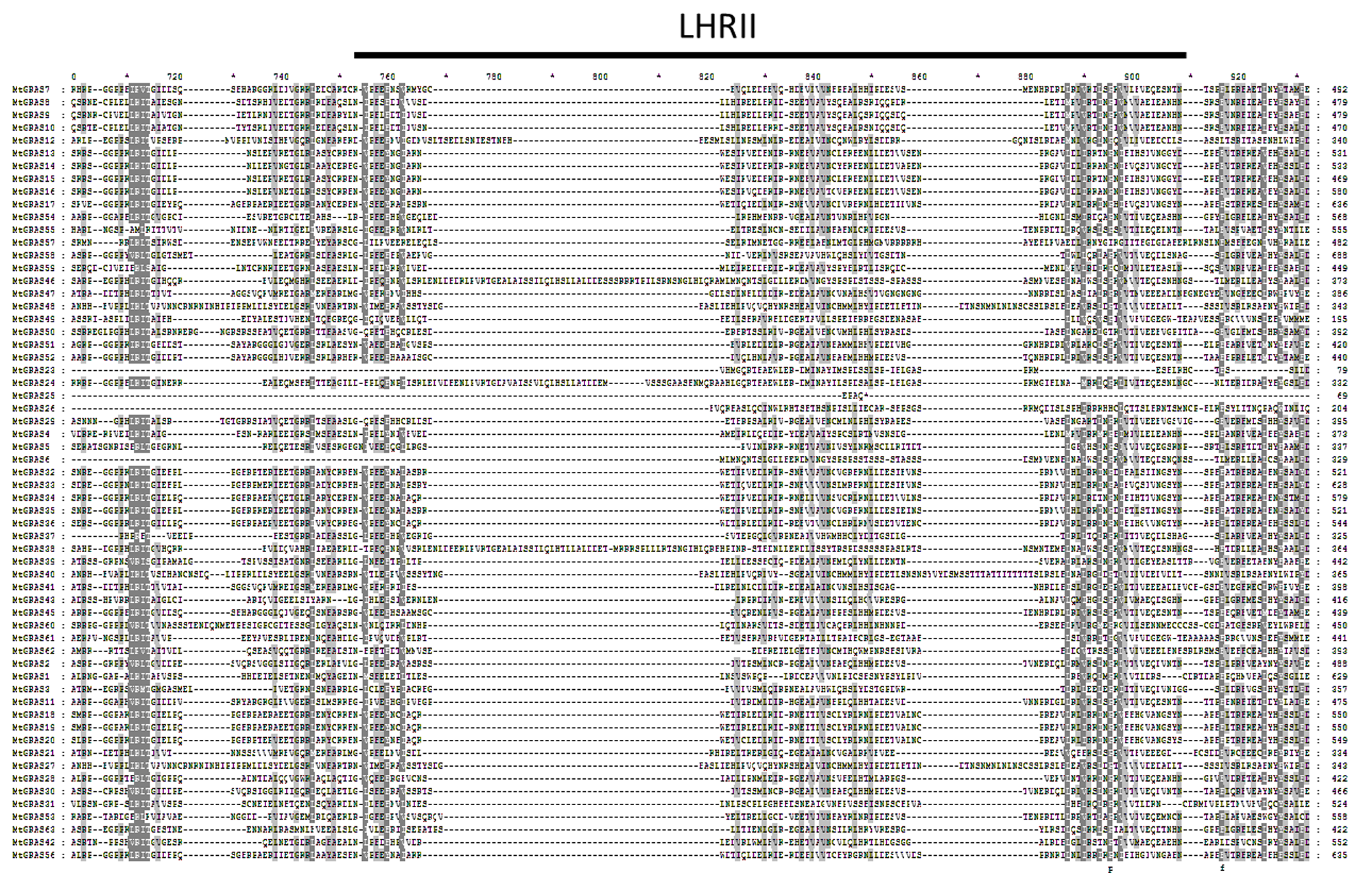

Supplement: S3 Fig — (TIF) [file pone.0185439.s003.tif]

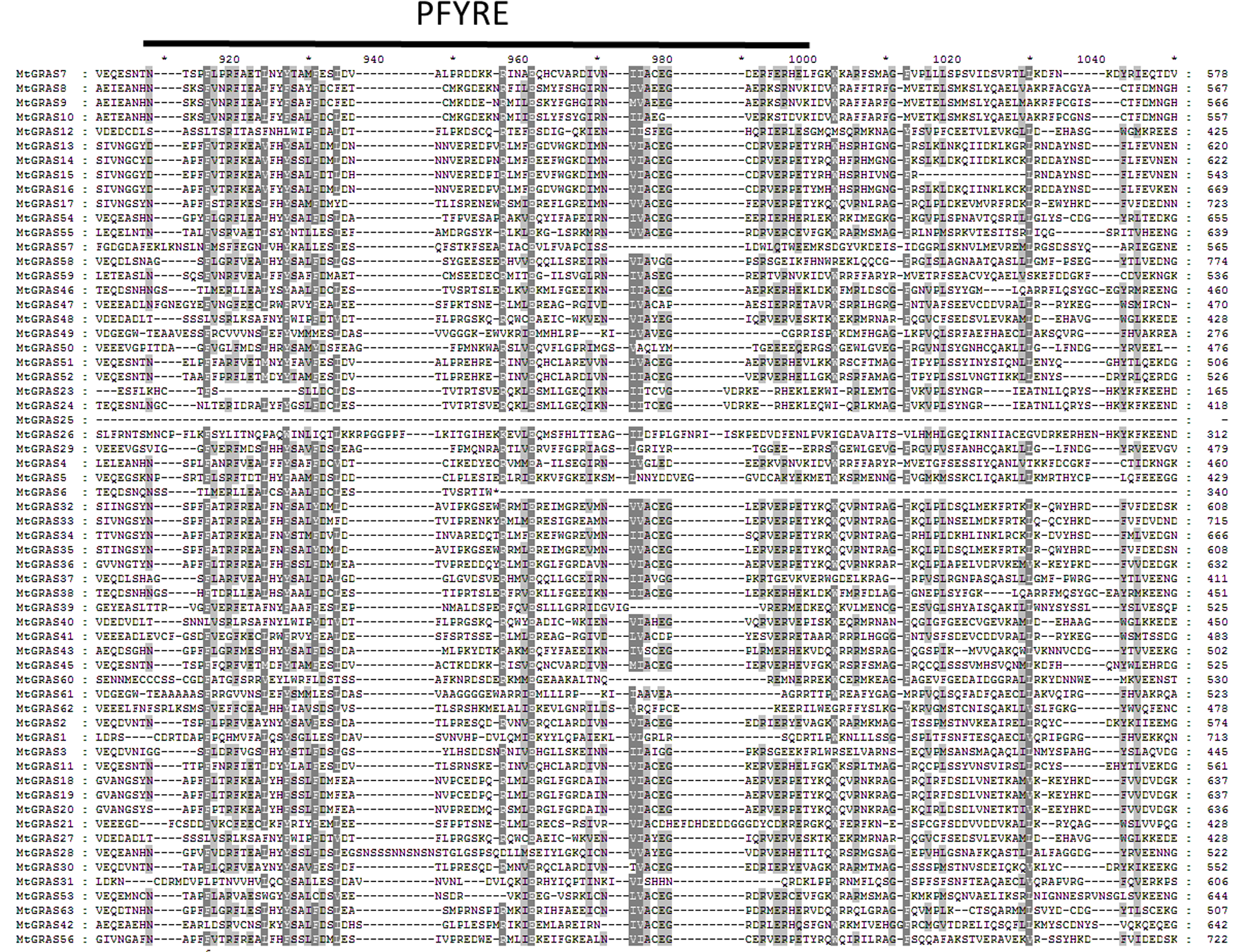

Supplement: S4 Fig — (TIF) [file pone.0185439.s004.tif]

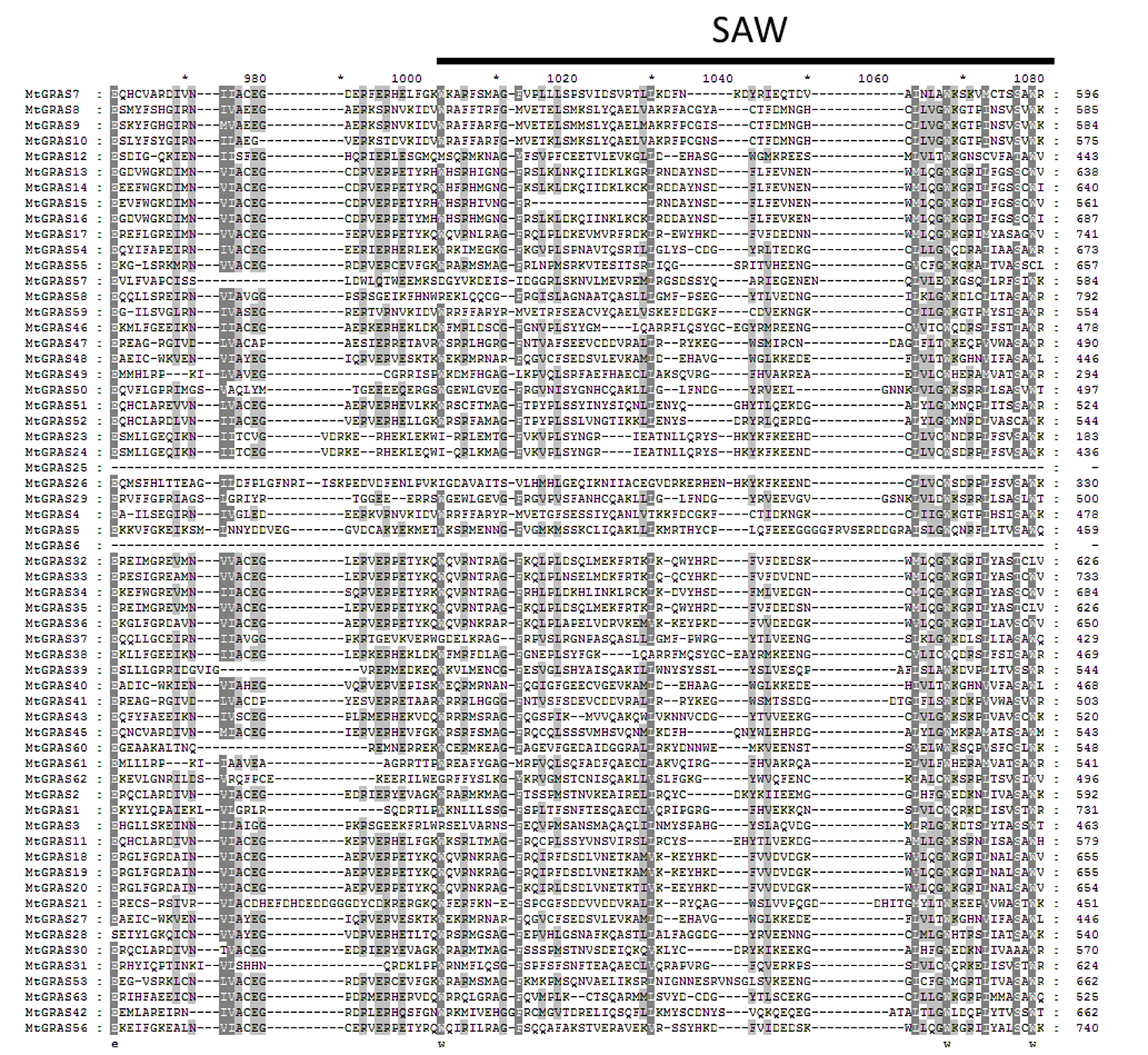

Supplement: S5 Fig — (TIF) [file pone.0185439.s005.tif]

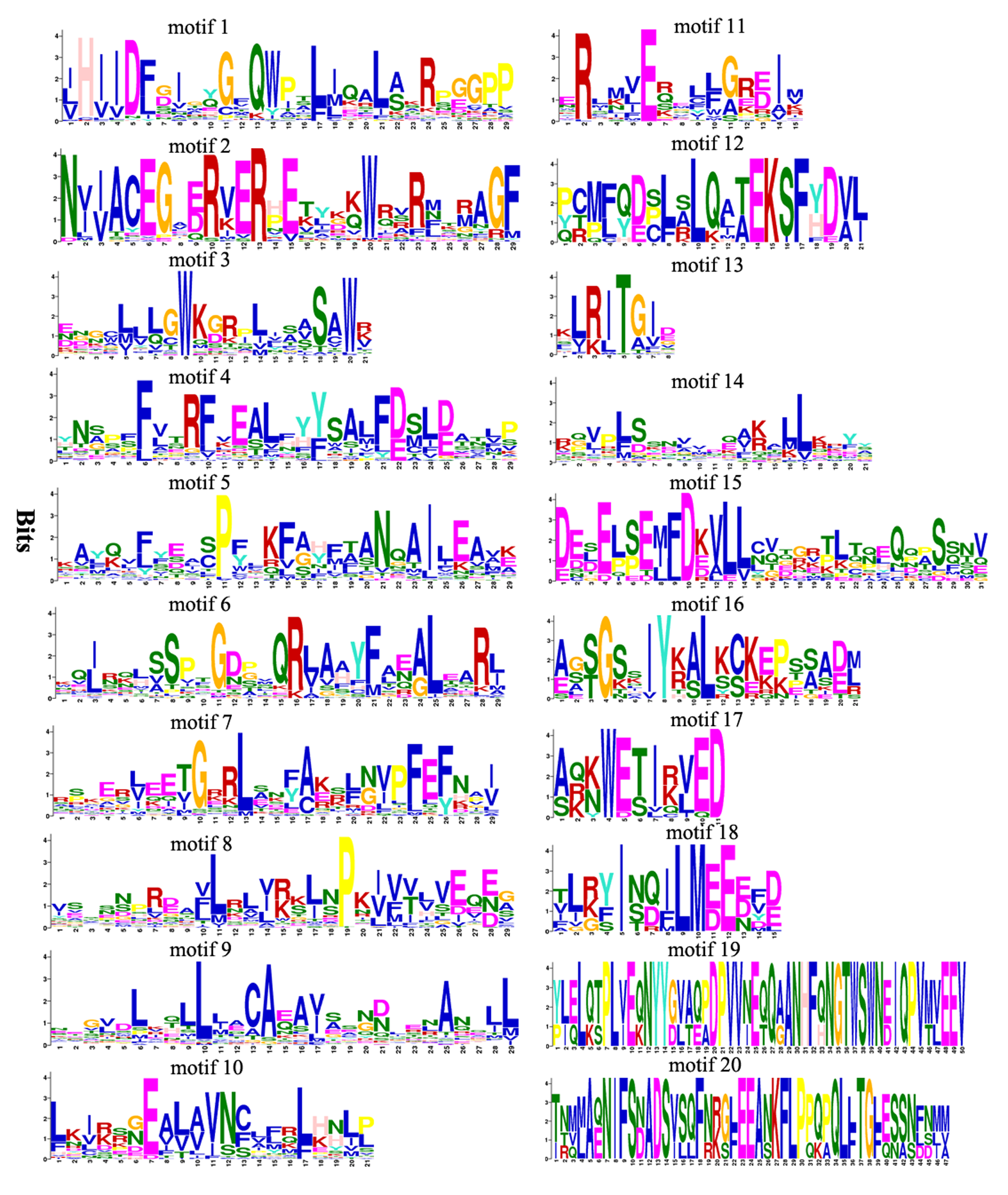

Supplement: S6 Fig — The logo represents conserved amino acids sequences in different motifs, and the heights of letters in the logo represent the frequency of amino acid at specific positions. (TIF) [file pone.0185439.s006.tif]

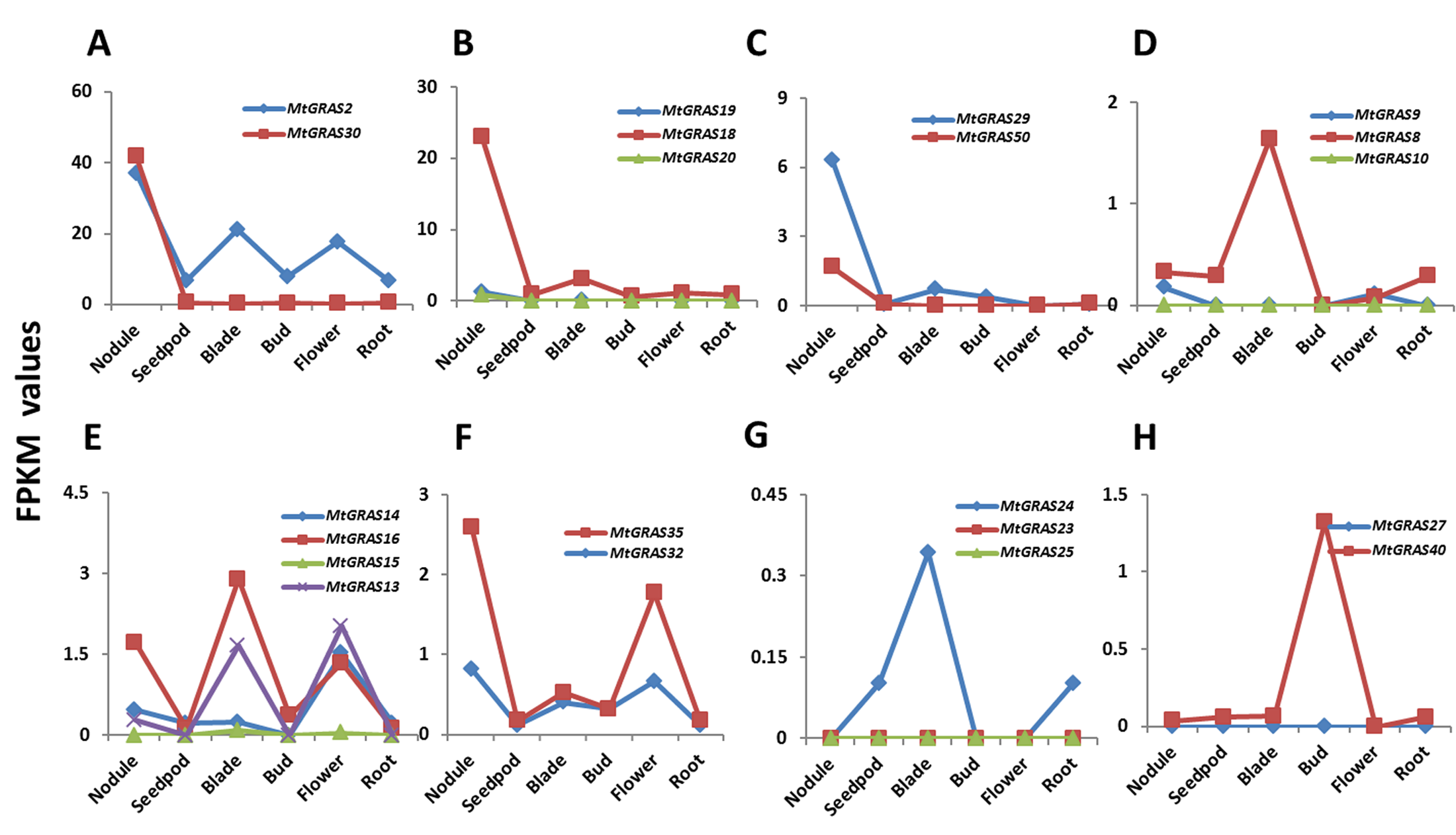

Supplement: S7 Fig — X-axis represents different tissues of M. truncatula. Y-axis shows the expression values (RPKM) obtained using RNA-seq data. (TIF) [file pone.0185439.s007.tif]
