# Supplementary material for: Genome-wide characterization of GRAS family genes in Medicago truncatula reveals their evolutionary dynamics and functional diversification
Source: PLoS One. 2017 Sep 25;12(9):e0185439. doi: 10.1371/journal.pone.0185439 (PMC5612761; doi:10.1371/journal.pone.0185439)
Supplement: S1 Table — (DOCX) [file pone.0185439.s008.docx]

| **Primer Name** | **sequence (5'-3')** |
| --- | --- |
| MtActin-F | CCACATGCCATCCTTCGTTT |
| MtActin-R | TGTCACGGACAATTTCCCG |
| MtGRAS32-F | GATGAAGACAGCAAATGGATG |
| MtGRAS32-R | AAAGTTAGAAAAAATGAGGAGTGAG |
| MtGRAS35-F | TTCAAGGTTGGAAGGGTCG |
| MtGRAS35-R | CACAAATGTCGGAAAGTTAGAAAAAA |
| MtGRAS37-F | CTCCAGCGAGAAAGCCGT |
| MtGRAS37-R | GCACATTGAAGCAAAAGCC |
| MtGRAS38-F | ACCATGTAGCTGCTTGTAATCTT |
| MtGRAS38-R | GTATCGCCGTCAGGGGAA |
| MtGRAS39-F | GGTATTAGGCAAAGCGGC |
| MtGRAS39-R | GAAAATCAGTCAAAGAGTAAAGTGAA |
| MtGRAS45-F | AATCATACCCAGAAATCACCG |
| MtGRAS45-R | GCTTCAAATCATTCCCATCG |
| MtGRAS46-F | TTGAAAAAGATATGGTTAATGGC |
| MtGRAS46-R | ATGAAGATGCTGGCGACG |
| MtGRAS47-F | GACGATACTCCTCACCTCCG |
| MtGRAS47-R | TTTTTCCATTCTAGCACCAATT |
| MtGRAS50-F | TACCACCCCACCCACTTC |
| MtGRAS50-R | TTTCTTCTTCATCATCTTGCTCTA |
| MtGRAS51-F | TTGCCACGGGAGCACAGA |
| MtGRAS51-R | CGCCTTCACACGCTACTAAAT |
| MtGRAS60-F | GATTTCGCCACTGGATTCTC |
| MtGRAS60-R | CCATTTTTCTCTCCTTTCGTTC |
| MtGRAS61-F | TTCTATTTTCACAACGAACCAA |
| MtGRAS61-R | TAACGATCCATGCAACGC |
