# Supplementary material for: Genome-wide characterization of GRAS family genes in Medicago truncatula reveals their evolutionary dynamics and functional diversification
Source: PLoS One. 2017 Sep 25;12(9):e0185439. doi: 10.1371/journal.pone.0185439 (PMC5612761; doi:10.1371/journal.pone.0185439)
Supplement: S2 Table — (DOCX) [file pone.0185439.s009.docx]

| **Gene symbol** | **Nodule_FPKM** | **Seedpod_FPKM** | **Blade_FPKM** | **Bud_FPKM** | **Flower_FPKM** | **Root_FPKM** |
| --- | --- | --- | --- | --- | --- | --- |
| *MtGRAS5* | 0 | 0 | 0 | 1.15613 | 0.11774 | 0 |
| *MtGRAS2* | 37.0833 | 6.78152 | 21.1475 | 7.95494 | 17.7391 | 6.78152 |
| *MtGRAS3* | 6.31185 | 0.097885 | 0 | 0 | 0 | 0.097885 |
| *MtGRAS4* | 0 | 0 | 0 | 0 | 0 | 0 |
| *MtGRAS6* | 2.08854 | 0.305386 | 1.19741 | 0.82763 | 0.505714 | 0.305386 |
| *MtGRAS12* | 0.089056 | 0 | 0.459526 | 1.85276 | 0.037737 | 0 |
| *MtGRAS13* | 0.277226 | 0 | 1.66888 | 0 | 2.03618 | 0 |
| *MtGRAS14* | 0.474028 | 0.218334 | 0.244596 | 0 | 1.52658 | 0.218334 |
| *MtGRAS15* | 0 | 0 | 0.094939 | 0 | 0.046779 | 0 |
| *MtGRAS16* | 1.72911 | 0.13614 | 2.89778 | 0.368953 | 1.35267 | 0.13614 |
| *MtGRAS17* | 13.466 | 4.39392 | 14.0419 | 4.01112 | 10.0336 | 4.39392 |
| *MtGRAS18* | 23.0686 | 0.930923 | 3.17609 | 0.688065 | 1.16787 | 0.930923 |
| *MtGRAS19* | 1.32476 | 0 | 0.061583 | 0 | 0.030344 | 0 |
| *MtGRAS20* | 0.850668 | 0 | 0 | 0 | 0 | 0 |
| *MtGRAS7* | 0.051564 | 0.554165 | 0 | 0 | 0 | 0.554165 |
| *MtGRAS8* | 0.334713 | 0.293651 | 1.64486 | 0 | 0.081047 | 0.293651 |
| *MtGRAS9* | 0.182865 | 0 | 0 | 0 | 0.116231 | 0 |
| *MtGRAS10* | 0 | 0 | 0 | 0 | 0 | 0 |
| *MtGRAS11* | 9.77078 | 3.71189 | 30.1879 | 6.59081 | 18.064 | 3.71189 |
| *MtGRAS21* | 0 | 1.82737 | 0.102359 | 2.47619 | 1.26088 | 1.82737 |
| *MtGRAS25* | 0 | 0 | 0 | 0 | 0 | 0 |
| *MtGRAS27* | 0 | 0 | 0 | 0 | 0 | 0 |
| *MtGRAS30* | 41.6991 | 0.538095 | 0.283673 | 0.457496 | 0.326138 | 0.538095 |
| *MtGRAS23* | 0 | 0 | 0 | 0 | 0 | 0 |
| *MtGRAS24* | 0 | 0.102577 | 0.344746 | 0 | 0 | 0.102577 |
| *MtGRAS26* | 0 | 0 | 0 | 0 | 0 | 0 |
| *MtGRAS28* | 62.7957 | 66.4631 | 64.0589 | 46.5138 | 38.8141 | 66.4631 |
| *MtGRAS29* | 6.3158 | 0.070779 | 0.713631 | 0.383637 | 0 | 0.070779 |
| *MtGRAS31* | 16.9999 | 3.79835 | 3.70813 | 4.70581 | 4.19334 | 3.79835 |
| *MtGRAS32* | 0.826366 | 0.120831 | 0.406095 | 0.327466 | 0.666982 | 0.120831 |
| *MtGRAS36* | 16.0689 | 6.07975 | 13.4532 | 9.53202 | 13.2021 | 6.07975 |
| *MtGRAS38* | 3.66071 | 1.12407 | 1.42717 | 5.6865 | 0.744573 | 1.12407 |
| *MtGRAS41* | 7.18185 | 7.11378 | 1.75328 | 9.63957 | 2.04192 | 7.11378 |
| *MtGRAS43* | 0 | 0.593219 | 0 | 0.45934 | 0.233895 | 0.593219 |
| *MtGRAS33* | 7.07687 | 1.75692 | 5.74936 | 5.01206 | 3.39434 | 1.75692 |
| *MtGRAS34* | 2.69034 | 3.39852 | 15.4049 | 3.68414 | 6.14738 | 3.39852 |
| *MtGRAS35* | 1.77475 | 0.054482 | 0.122088 | 0 | 1.11301 | 0.054482 |
| *MtGRAS37* | 8.55564 | 17.7118 | 13.1016 | 8.72745 | 21.6119 | 17.7118 |
| *MtGRAS39* | 9.39603 | 11.2012 | 23.069 | 24.8372 | 9.77416 | 11.2012 |
| *MtGRAS40* | 0.039765 | 0.061054 | 0.068398 | 1.3238 | 0 | 0.061054 |
| *MtGRAS45* | 63.4184 | 10.2297 | 26.3031 | 16.0329 | 11.9058 | 10.2297 |
| *MtGRAS48* | 0 | 0 | 0 | 0 | 0 | 0 |
| *MtGRAS52* | 4.03871 | 7.94814 | 6.12555 | 5.8052 | 7.53003 | 7.94814 |
| *MtGRAS46* | 4.85021 | 2.53615 | 3.62707 | 2.92479 | 0.565934 | 2.53615 |
| *MtGRAS47* | 13.6915 | 5.54711 | 4.82428 | 14.6377 | 5.35846 | 5.54711 |
| *MtGRAS49* | 0 | 0 | 0 | 0 | 0 | 0 |
| *MtGRAS50* | 1.68356 | 0.095733 | 0 | 0 | 0 | 0.095733 |
| *MtGRAS51* | 5.13805 | 0.695174 | 2.53519 | 1.02764 | 3.90664 | 0.695174 |
| *MtGRAS53* | 107.799 | 15.6498 | 18.226 | 5.79743 | 55.0013 | 15.6498 |
| *MtGRAS57* | 1.96799 | 0 | 0 | 0 | 0 | 0 |
| *MtGRAS54* | 0 | 0 | 0 | 0 | 0 | 0 |
| *MtGRAS55* | 0.092826 | 0.071258 | 0 | 0.386236 | 0.078668 | 0.071258 |
| *MtGRAS58* | 6.75216 | 1.78736 | 1.37939 | 9.47258 | 3.83682 | 1.78736 |
| *MtGRAS59* | 0.244774 | 0 | 0 | 0 | 0.082977 | 0 |
| *MtGRAS60* | 22.5065 | 0.123189 | 0 | 0 | 0 | 0.123189 |
| *MtGRAS61* | 16.2861 | 9.08706 | 18.231 | 9.01378 | 56.1924 | 9.08706 |
| *MtGRAS62* | 0.109879 | 0 | 0 | 0 | 0 | 0 |
| *MtGRAS63* | 0.453451 | 0.087024 | 0 | 0 | 0.144109 | 0.087024 |
| *MtGRAS1* | 45.0987 | 12.5183 | 25.7852 | 0 | 10.8156 | 12.5183 |

Note: FPKM, Fragments Per Kilobase of exon per Million fragments mapped.
