# Supplementary material for: Genome-wide characterization of GRAS family genes in Medicago truncatula reveals their evolutionary dynamics and functional diversification
Source: PLoS One. 2017 Sep 25;12(9):e0185439. doi: 10.1371/journal.pone.0185439 (PMC5612761; doi:10.1371/journal.pone.0185439)
Supplement: S3 Table — (DOCX) [file pone.0185439.s010.docx]

| **Groups** | **Gene symbol** | **Expression pattern** | **Homolog gene** | **Expression pattern** |
| --- | --- | --- | --- | --- |
|  |  |  |  |  |
| PAT1 | *MtGRAS2* | expressed, nodule highest | *MtGRAS30* | expressed, nodule highest |
|  |  |  |  |  |
| SCR | *MtGRAS9* | low expressed, nodule highest | *MtGRAS8* | low expressed, blade highest |
|  |  |  |  |  |
| LISCL | *MtGRAS16* | expressed, nodule highest | *MtGRAS14* | expressed, flower highest |
|  |  |  |  |  |
| LISCL | *MtGRAS16* | expressed, nodule highest | *MtGRAS13* | expressed, flower highest |
|  |  |  |  |  |
| LISCL | *MtGRAS19* | low expressed, nodule highest | *MtGRAS20* | low expressed, nodule specific |
|  |  |  |  |  |
| SCL3 | *MtGRAS24* | low expressed, blade highest | *MtGRAS25* | no expressed |
|  |  |  |  |  |
| LISCL | *MtGRAS32* | low expressed, nodule highest | *MtGRAS33* | expressed, nodule highest |
|  |  |  |  |  |
| SCL3 | *MtGRAS38* | expressed, nodule higheset | *MtGRAS46* | expressed, nodule highest |
|  |  |  |  |  |
| HAM | *MtGRAS49* | no expressed | *MtGRAS61* | expressed, nodule highest |
|  |  |  |  |  |
